# Declining Ecosystem Respiration Linked to Nitrogen Deposition: Insights From a 26‐Year FLUXNET Record

**DOI:** 10.1111/gcb.70849

**Published:** 2026-04-13

**Authors:** Michiel K. van der Molen, Marnix van de Sande, Michiel in 't Zandt, Tori Saccomandi, Sophie L. Baartman, Hong Zhao, Jordi Vilà‐Guerau de Arellano

**Affiliations:** ^1^ Meteorology and Air Quality Group Wageningen University Wageningen the Netherlands; ^2^ Soil Biology Group Wageningen University Wageningen the Netherlands; ^3^ MSc Environmental Sciences Wageningen University Wageningen the Netherlands; ^4^ Department of Ecology University of Innsbruck Innsbruck Austria

**Keywords:** acidification, carbon sequestration, ecosystem respiration, FLUXNET, nitrogen, soil biology

## Abstract

Long‐term carbon flux measurements at the FLUXNET site Loobos, a Pine forest in the Netherlands, reveal a counter‐intuitive decline in total ecosystem respiration (*TER*) by tens of percents between 1997 and 2021. This trend cannot be explained by temperature variability or methodological changes alone. Instead, our findings point to a biogeochemical mechanism: despite a doubling of soil organic matter stocks, ecosystem respiration appears limited by decomposition rates rather than substrate availability. Soil incubation experiments indicate that microbial activity is limited by substrate quality and strongly acidic conditions (pH = 2.9), associated with large nitrogen deposition. Glucose addition experiments confirm the presence of an active microbiome, but its activity is suppressed under the present acidic soil conditions. These results raise concerns about ecosystem health under conditions of nitrogen deposition and the long‐term sustainability of the observed carbon sink. Loobos may serve as an early indicator of broader ecosystem responses to environmental disturbances, as similar negative *TER* trends have been observed at other long‐term FLUXNET sites. To advance understanding of the global carbon cycle, it is essential that observed flux trends are attributed and corroborated by changes in carbon and nitrogen stocks, and that models are continuously confronted with observational data. We therefore discuss the need of periodically measuring pH as soil acidification can be a limiting factor and suggest the need to introduce this variable in model representations of TER near regions sensitive to nitrification.

## Graphical abstract

Ecosystem respiration in aging forests is expected to grow to a high level. Counter‐intuitively, in the Loobos (NL) forest, we find respiration to decrease by tens of percents over the last 26 years. This is linked to acidification of the soil due to nitrogen deposition. Interestingly, we find decreasing trends for multiple aging FLUXNET forest sites. This may be associated to large‐scale pollution and disturbances of ecosystems. While suppressed respiration contributes to carbon storage, it also raises questions about ecosystem resilience.

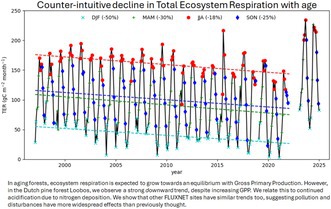

## 1 Introduction

FLUXNET (Baldocchi et al. 2001) and its regional components (e.g., AmeriFlux (Novick et al. 2018), AsiaFlux (Yamamoto et al. 2005), ChinaFlux (Yu et al. 2006), OzFlux (Beringer et al. 2016), and the Ecosystem Thematic Center in the Integrated Carbon Observing System (ICOS) in Europe (Heiskanen et al. 2021)) were born at the end of the 20th century. This network of eddy covariance stations is growing in temporal scale and spatial resolution. Some sites approach the 30‐year period of record. Interestingly, the majority of sites report a net carbon (C) sink on an annual basis (*cf*. Friend et al. 2007; Phillips et al. 2009; Gough et al. 2008; Grant et al. 2009). The observed carbon uptake is often reduced when climate extremes such as drought or heatwaves occur (*cf*. Gharun et al. 2025). A comprehensive network overview is lacking, but the observed sinks are generally in agreement with multi‐approach‐based analyses, reporting a global land‐sink that is vulnerable to climate disturbances (Friedlingstein et al. 2025; van der Woude et al. 2023). However, Randerson et al. (2025) make the point that the land sink reported by the Global Carbon Project (Friedlingstein et al. 2023, 2025) based on forward and inversion models is larger than the changes in biomass seen with remote sensing techniques. Hence, we need to allocate the sinks observed at ecosystem sites in terms of carbon stock changes and explain the changes in terms of ecological and biogeochemical processes.

According to classic ecosystem dynamics theory, ecosystem carbon uptake via photosynthesis and release via autotrophic and heterotrophic respiration and lateral export progress towards an equilibrium as the ecosystem matures (Odum 1969). This occurs as biomass increases over time and the associated overturning of biomass (respiration) balances the initially larger uptake. Nuancing this classic theory, Luyssaert et al. (2008) and Curtis and Gough (2018) show that mature, old‐growth forests may still sequester carbon as ecosystem complexity, light interception and nitrogen use efficiency increase relatively fast in older forests, while carbon from dead trees is released slowly. Sustained carbon sinks in mature, undisturbed ecosystems are characterised by fast seasonal uptake by photosynthesis and slow, decadal‐scale release by decomposition of stable soil and litter carbon pools.

Ecosystem disturbance may impact the balance between uptake and release of carbon. Environmental disturbances include carbon dioxide fertilisation, warming (in temperate and arctic regions), droughts or floods, diseases and pests (Patacca et al. 2023) and nitrogen fertilisation (Fleischer et al. 2013; Durand et al. 2021; Chen et al. 2024). Nitrogen (N) deposition in N‐limited environments may lead to increased productivity in terrestrial ecosystems. This N‐induced global carbon sink is estimated at 177 ± 65 Tg C/year (Schulte‐Uebbing and de Vries 2018). Next to this effect on primary productivity, an analysis of several N addition experiments to soils also showed that N deposition can suppress soil microbial activity by lowering the pH, which ultimately reduces heterotrophic respiration (Janssens et al. 2010; Xiao et al. 2024). Small disturbances may lead to temporary changes in the carbon balance, eventually followed by compensating uptake in successive years (van der Molen et al. 2011; van der Woude et al. 2023). However, large, repeated or continued ecosystem disturbances may lead to increased tree mortality, changes in biodiversity and reduced ecosystem stability (Allen et al. 2010). In Europe, an increasing disturbance pressure is already associated with a negative trend in the forest carbon sink (Senf and Seidl 2021; Patacca et al. 2023; Migliavacca et al. 2025). This theoretical perspective emphasises the strong coupling of an ecosystem's carbon sink with the presence of environmental disturbances, which eventually affect the ecosystem's health and stability, feeding back on the long‐term sustainability of the carbon sink.

Here, we present the results from our long‐term flux site Loobos (NL‐Loo) as an example of a (near) mature forest site that acts as a net carbon sink. Substantial carbon uptake has been measured at the site since the measurements started in 1997 as one of the first FLUXNET sites (Zhao et al. 2026). Over the years, the observed carbon sink has increased (Net Ecosystem Exchange (*NEE*) becomes more negative), particularly in winter. Part of the increasing sink can be explained by a large negative trend in the total ecosystem respiration (*TER*) in the order of tens of percents (Section 3.1). As the forest was planted on bare sandy soil that did not contain any carbon, the current presence of biomass and soil organic matter implies that the carbon pools in the ecosystem have been increasing over time. Based on ecosystem development theory (Odum 1969), the ecosystem respiration is thus expected to increase with the size of above‐ground biomass and soil carbon pools, but this is clearly not the case at our site.

We hypothesise that the observed decrease in ecosystem respiration at Loobos is either (i) related to methodological developments at the site, because over the period 1997 to 2025 the top of the canopy grew towards the eddy covariance system, reducing its effective height, a phenomenon common to long‐term forest sites, or (ii) related to the large nitrogen deposition at the site (Section 2.1) and the associated soil acidification. The nitrogen availability has likely stimulated tree growth and needle production, increasing gross primary productivity (*GPP*). However, the nitrogen‐induced soil acidification may have resulted in an even slower decomposition of the needles that are already acidic and difficult to decompose. If acidification would explain the declining trend in *TER* and the associated increasing carbon sink, the question arises whether Loobos is indeed a healthy ecosystem and whether the carbon sink capacity can be sustained in the longer term. In that sense, the situation at Loobos could be a whistleblower for potential severe effects of environmental disturbances, in particular atmospheric nitrogen deposition.

To this end, we explore possible methodological and biogeochemical explanations behind the decreasing *TER* through data analysis of flux data, accompanied by the results from soil inventories, chamber measurements of soil respiration at the site and laboratory‐based soil respiration experiments. While the negative effects of nitrogen‐induced acidification on soil decomposition are well‐known (Janssens et al. 2010; Xiao et al. 2024), as far as we know, we are the first to combine an assessment of the long‐term negative *TER* trend with soil respiration experiments in a forested ecosystem flux site exposed to severe nitrogen deposition.

Putting our results in a larger perspective, we show that Loobos is not the only FLUXNET site with a long period of record and a decreasing *TER* (Section 3.1). Thus, the analysis that we show here is most likely also relevant for other sites that are under the influence of anthropogenic ecosystem disturbances. Papale et al. (2015), Falge et al. (2017), Baldocchi (2020), Migliavacca et al. (2021) and Villareal and Vargas (2021) argue that FLUXNET and its regional contributors achieve a satisfactory coverage of ecosystem types and climate zones. However, arguably, the majority of the FLUXNET sites (https://fluxnet.org/sites/site‐list‐and‐pages/?view=map, last access 11 August 2025) are located in more densely populated regions, which may be exposed to more than average anthropogenic disturbances and environmental pollution, such as nitrogen deposition. This emphasises that environmental (and climatic) disturbances may be common at FLUXNET sites.

After quantifying the magnitude of the *TER* trend, we pose the following research questions:

1. Do methodological causes offer a potential explanation for the observed trend?
2. How has soil organic matter changed over the period of record?
3. Can the observed trend be explained by biogeochemical processes?
4. What implications do the answers to these questions have for the interpretation of the carbon sink and the health of the ecosystems at Loobos and other FLUXNET sites?

Section 2 presents our methodology. Section 3 systematically presents the results from the long‐term analysis to the laboratory experiments relating pH soil acidification with CO_2_ respiration. In Section 4, we discuss a strategy for better detecting and accounting for environmental disturbances at FLUXNET sites.

## 2 Materials and Methods

In this section, we first describe our Loobos site and the flux measurements (Section 2.1), then we describe how we use Arrhenius functions to disentangle climate effects and the stability of the soil carbon pool (Section 2.2). Third, we describe how we collected soil samples and performed a series of laboratory incubation experiments (Section 2.2) to quantify soil respiration. The Supporting Information describes in more detail how we quantified the trend in *TER*, the comparison with other FLUXNET sites, the distribution of *TER* over autotrophic and soil respiration, the results of soil inventories and the laboratory experiments. All Loobos data used in this study are available via Zhao (2025), van der Molen et al. (2026a, 2026b, 2026c, 2026d, 2026e) and van der Molen, van de Sande, et al. 2026.

### 2.1 Site Description

We studied a pine ( *Pinus sylvestris* L.) forest (Loobos, NL‐Loo) in the Netherlands (52.166447° N, 5.74355° E, Koppen climate zone Cfb, marine west coast), which was planted as a monoculture in 1911 on bare, wind‐blown, mineral sandy soil. With the mineral sandy soil having a low water‐holding capacity and nutrient content, the trees only grew slowly at first, until in the 1960's intensive livestock farming was introduced upwind in the dominant wind direction and subsequent nitrogen deposition (38 and 21 kg N·ha^−1^·year^−1^ in 1990 and 2023, CLO 2025, Melman et al. 2025; van der Molen, Snellen, et al. 2026) caused enhanced forest growth. Zhao et al. (2026) describe how the above‐ground biomass has grown from 91 to 173 t C·ha^−1^, based on forest inventories between 1996 and 2025, and the *NEE* has become more strongly negative from −350 to −550 g C·m^−2^·year^−1^. A thick organic layer has formed on top of the mineral soil (Zhao et al. 2026, Appendix S4). The forest ecosystem has never been managed, at least not since the installation of the flux measurement site, and as far as we know not before either. Some tree cores taken from trees around the site date back to the 1920s, some others to the 1960s, suggesting there has been some regeneration. The site has been exposed to self‐thinning due to storm and snow damage to the treetops (Zhao et al. 2026).

A first flux tower became operational for CO_2_ flux measurements in 1997, which was replaced by a second one in 2021. The site, its geology and history, the meteorological and eddy covariance measurements and ancillary observations including forest inventories are extensively described in Zhao et al. (2026) and van der Molen, Snellen, et al. (2026). Appendix S1 describes the derivation of *TER* trends and its sensitivity to footprint and the u_*_ threshold value in detail. In addition, in Appendix S2, we made a comparison to flux measurement sites in other ecosystems to see whether a general trend in *TER* can be observed elsewhere as well. We focused on temperate and boreal forest ecosystem sites from the FLUXNET, AMERIFLUX and ICOS‐ETC datasets with a long (5+ years) period of record. A list of included sites and the procedure to assess the trends in *TER* is included. Appendix S3 describes chamber measurements at the site, which we use to distinguish between soil respiration and autotrophic respiration. Effect of temperature changes on TER.

Over the period of record, the temperature has changed due to global warming, which could cause a trend in *TER* over time. Lloyd and Taylor (1994) describe the dependence of soil respiration on temperature using an Arrhenius‐like exponential function:

(1) $$\mathrm{TER}=R_{10}\;e^{\left(\frac{E_{a}}{T_{0}\cdot R^{*}}\left(1-\frac{T_{0}}{T_{\text{soil}}}\right)\right)}$$

where $R_{10}$ (μmol·m^−2^·s^−1^) is the reference respiration at a reference temperature of *T*_*o*_ = 283.15 K under no water stress condition, $E_{a}$ (J·mol^−1^) is the activation energy parameter controlling the temperature sensitivity, R* the universal gas constant (8.314 J·K^−1^·mol^−1^) and $T_{\text{soil}}$ is soil temperature (K), in this case the temperature of the organic layer on top of the mineral soil. In principle, the *R*_10_ value can be dependent on soil moisture (Jacobs et al. 2007). Because the availability of soil moisture data appeared to often be limiting the analysis, we decided to only use data from October to March, arguing that in these periods droughts do not occur and the organic layer on top of the mineral soil is always humid but not saturated. Additionally, we fitted the parameters using 6 months of daily average data, because on a monthly or seasonal scale, the temperature variation was often too small to constrain *E*_a_ well. We fitted the above function to estimates of *TER* to derive annual values for *R*_10_ and *E*_a_. In Section 3.2, we use the temporal trends in *R*_10_ and *E*_a_ to infer if the trend in *TER* could be explained by changes in temperature or reference respiration rate.

### 2.2 Soil Incubation Experiments

#### 2.2.1 Soil Sampling and Sample Preparation

Soil samples were collected from organic matter‐rich topsoil layers at the Loobos forest on the 15th and 16th of April 2025. Sample locations were based on the previous ICOS conform soil inventory using 20 locations in 2024 (van der Molen, Snellen, et al. 2026). Ten locations were chosen within a 760 m radius from the tower (Appendix S5). At each location, three soil samples were taken from a root‐free surface at a randomly selected spot within a 2 m radius from the central coordinates of the respective sample location. For each sample, a 15 cm × 15 cm × 10 cm metal square cutter was inserted, and the organic layer was collected, while the mineral soil was not included. All samples were stored at 4°C until further processing. Data are archived in van der Molen, van de Sande, et al. (2026).

In the laboratory, soil samples were manually shaken to separate and remove detritus and roots. Next, the soil samples were sieved at 5 mm to remove large organic matter particles, and the remaining soil was stored at 4°C until further analysis.

#### 2.2.2 Moisture Content and Water Holding Capacity

The moisture content of the field‐fresh soils was determined by air‐drying at 105°C for 24 h in a forced ventilation oven. For the respiration experiment, all soils were corrected to 60% of the water‐holding capacity (WHC). For this soil type, the WHC was unexpectedly high, leading to challenges in accurately determining the WHC. Therefore, the average between the last and second to last water addition for the determination of 100% WHC was considered as the true 100% WHC for these samples. For field‐fresh soils with a WHC below 60%, demineralised water was added until 60% WHC was reached. Field‐fresh soil samples over 60% WHC were air‐dried at room temperature until WHC < 64%. For further details on moisture content and WHC, see Appendix S5.

#### 2.2.3 Substrate and Lime Treatment

For each sampled location, four bottles were prepared with the following treatments: control, substrate, lime and substrate & lime. For all bottles, fresh soil was added according to the equivalent of 50 g of dry soil for each SP‐I location. For the glucose addition, a concentration of 3 g per kg dry soil was added. For the lime addition, a 20% w/w dose of calcium carbonate per dry soil weight equivalent was added. For detailed information, see Appendix S5.

#### 2.2.4 Measurements of pH, C:N and CO_2_

Soil pH was measured at the start and the end of the respiration experiment using a 0.01 M CaCl_2_ solution. The carbon and nitrogen contents and C:N ratio were determined using a FlashSmart Elemental Analyzer (Thermo Scientific, Germany) following manufacturer instructions.

Soil respiration data were calculated in μg CO_2_ per kg dry soil per hour. For this calculation, Equation (2) was used:

(2) $$E_{\text{bottles}}=\frac{V_{\mathrm{tot}}\times C_{\text{flask}}-V_{\mathrm{int}}\times C_{\mathrm{bg}}}{M_{\mathrm{dry}}\times V_{m}}\times \frac{M_{C}}{t}$$

where *E*_bottles_ is the emission rate of the experiment bottles (μg dry soil·hour^−1^), *V*_tot_ is the total bottle volume (L), *C*_flask_ is the concentration measured in the bottle (ppmV), *V*_int_ is the headspace inside the monitor (L), *C*_bg_ is the concentration measured in the background gas—compressed air (ppmV), *M*_*C*_ = 12 g·mole^−1^ is the molar weight of carbon, *M*_dry_ is the total dry weight of the soil added to the bottle in kg, *V*_m_ is the molar volume of gas under reference conditions (L mole^−1^) and *t* is the incubation time (h).

## 3 Results

### 3.1 *TER* Trends

**FIGURE 1:**
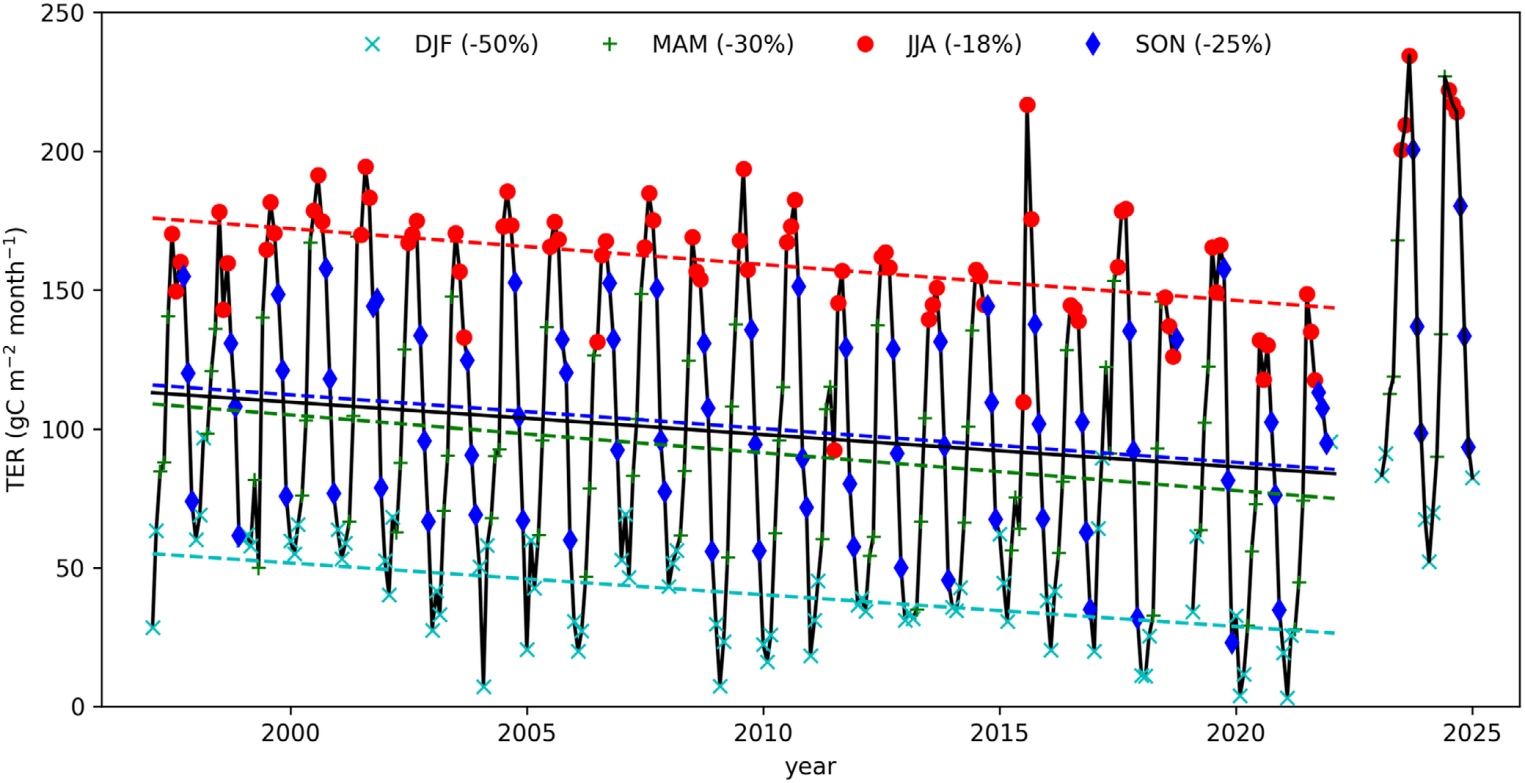
Trend in TER per season. The markers show monthly sums. The colours combine the months into seasons as indicated in the text within the figure. The dashed lines indicate the linear regression lines of seasonally mean TER. The percentages shown in the legend indicate the change in TER from 1997 to 2021 relative to 1997. From 1997 to 2021, the TER from NEE partitioning is shown (Zhao et al. 2026). The post 2021 data represent TER from the ICOS FLUXNET half‐hourly product from 2023 to 2024 (van der Molen et al. 2026b).

Figure 1 shows the estimated total ecosystem respiration (*TER*). A negative trend is observed in all seasons over the period 1997 to 2021. The trends in *TER* over the 26‐year period are in the order of tens of percents of the *TER* in 1997 (see also Table S1). There are seasonal and interannual variations superimposed on the trend, and towards the end of the period of record, the variability increases. This increase in variability is possibly as a result of more frequent climate extremes, such as droughts and heatwaves in 2003, 2013 and 2018. With the installation of the second tower after 2021, the *TER* estimates appear larger than before.

While *TER* decreases from 1350 g C·m^−2^·year^−1^ in 1997 to 1015 g C·m^−2^·year^−1^ in 2021, a decrease of 335 g C·m^−2^·year^−1^, in contrast, *GPP* increases from 1567 in 1997 to 1904 g C·m^−2^·year^−1^ in 2021, an increase of 337 g C·m^−2^·year^−1^ (83% in winter, 3% in spring, 8% in summer and 40% in fall). On an annual basis, *NEE* = *TER*—*GPP* increases from −217 g C·m^−2^·year^−1^ to −889 g C·m^−2^·year^−1^.

Needle and total litter fall, measured from 2004 to 2015 in 24 trays of 0.5 m^2^, were 300.7 (± 45.5) and 494.9 (± 62.5) g·m^−2^·year^−1^ and were not subject to a significant trend.

In Appendix S1, we explore several possible instrumental or data processing explanations of the estimated trend. One relevant fact is that the canopy top (15.3 m in 1996) grew 16 cm·year^−1^, reducing the effective height (*z*‐*d*) of the eddy covariance system at *z* = 27 m from 16.8 m in 1997 to 14.3 m in 2021, a change Δ(*z*‐*d*) = 2.5 m (Appendix S1). This could affect the flux observations via changes in (1) the eddy size distribution, (2) the footprint area and (3) the u_*_ selection criteria. In Appendix S1, we provide arguments and observational evidence suggesting these issues cannot explain the estimated trend in *TER*. We do acknowledge the remarkable change in *TER* after changing to the second tower data in 2023, which presumes that some instrumental or height‐related issue still influences the data, while we remain speculative at this point. Since the period of the new record is still so short (2 years), we remain inconclusive about this period and consequently we focus on the period characterised by the longest and more consistent record (from 1997 to 2021).

**FIGURE 2:**
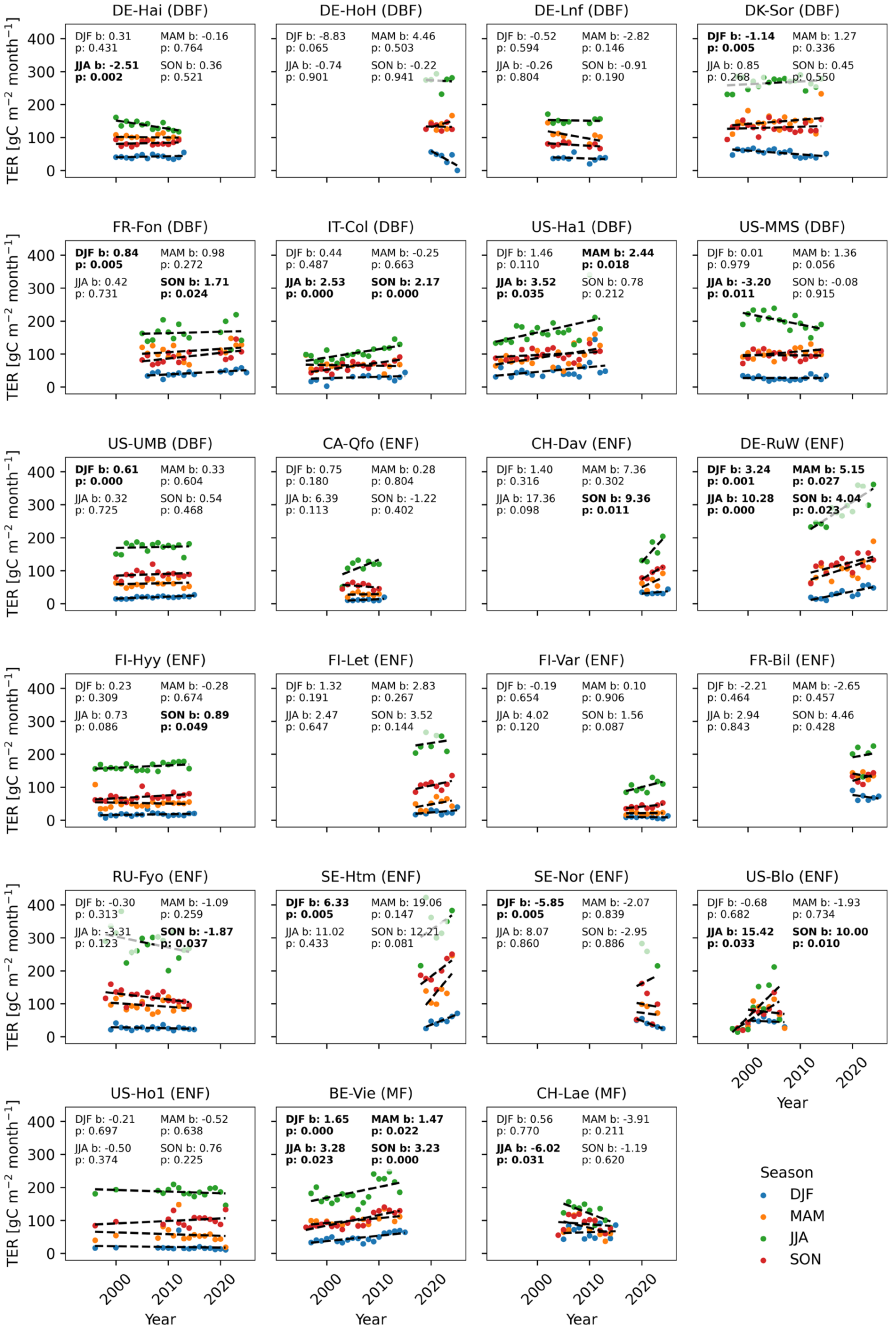
Seasonal mean TER across several temperate forests with long measurement histories, shown in units gC m^−2^ month^−1^ to match the units of Figure 1. The sub‐plots show TER for each station included in the analysis, with different colours and fitted slopes for each season: DJF—December, January and February; MAM—March, April and May; JJA—June, July and August; SON—September, October and November. The plant functional type (PFT) of the respective site is included in parentheses; DBF indicates deciduous broadleaf forests, ENF indicates evergreen needleleaf forests and MF indicates a mixed forest.

Before investigating the biogeochemical explanations, we first place the negative *TER* trend in a broader FLUXNET perspective. The decreasing trend in estimated *TER*, as presented above for the case of Loobos, is corroborated by several other long‐term measurement stations in temperate and boreal forest ecosystems. Figure 2 shows seasonal average *TER* for several other measurement stations located in temperate or boreal forests (see Appendix S2 for a detailed description of the data). Stations Hainich (DE‐Hai), Soroe (DK‐Sor), Morgan Monroe State Forest (US‐MMS), Fyoderovskoje (Ru‐Fyo), Norunda (SE‐Nor) and Laegern (CH‐Lae) show a negative trend in *TER* during one of the seasons. Notably, except for Norunda, these sites are all mature (> 100 years old) forests. It is interesting to note that the Morgan Monroe State Forest and the Hainich sites have not been subject to management since 1965 (Roman et al. 2015; Tamrakar et al. 2018). The Fyoderovskoje site (Ru‐Fyo) is a wet, old‐growth *Picea abies* forest in Russia (Kurbatova et al. 2008). The Norunda site has been observing a mature (100 years old) pine forest until a clearcut in 2022 (Lindroth et al. 2018; Petersen et al. 2025), which could very well have confounded the observed downward trend in TER. The Swiss Laegern site has a mixed *Picea abies* (125–205 years old) and *Fagus sylvatica* (70–175 years old) forest (Eugster et al. 2007).

### 3.2 The Soil Organic Layer and Temperature Response

Above we have shown that a negative trend in *TER* occurred in Loobos over the 26 period of record. Appendix S3 shows that *TER* is composed of an average of 69% soil respiration and 31% above‐ground autotrophic respiration. This implies that the negative trend in *TER* cannot be explained by a potential negative trend in above‐ground autotrophic respiration alone. Zhao et al. (2026) show that the above‐ground biomass has increased from 121 ton dry matter·ha^−1^ in 2000 to 173 ton·ha^−1^ in 2025. With increasing heart wood, the autotrophic respiration rate per mass wood may decrease. Nevertheless, from a mathematical viewpoint, the majority of the change in *TER* must have been caused by a change in soil respiration.

In 2009, most (67%) of the organic matter in the soil was contained in the organic layer on top of the mineral soil (Appendix S4). A comparison with the 2024 soil inventory shows that this organic layer has compacted and doubled in mass. Unfortunately, the carbon and nitrogen content analysis over the 2024 inventory has not yet been completed. However, considering that the C:N ratio (partly based on lab samples) has not changed from around 25, this may suggest that the carbon pool in the organic matter layer has increased. Assuming a constant carbon mixing ratio (kg C·kg^−1^ soil), the organic layer carbon pool would have doubled from 4.7 kg C·m^−2^ in 2009 to 9.4 kg C·m^−2^ in 2024, an increase of 313 g C·m^−2^·year^−1^. This could explain a large fraction of *NEE*, which is typically around 500 g C·m^−1^·year^−1^ (Zhao et al. 2026). However, the accumulation of 313 g C·m^−2^·year^−1^ must be interpreted with large uncertainty margins because of the different measurement locations, sampling protocols and analysis methods. While we cannot provide conclusive evidence yet, and we may not be able to provide it later due to differences in soil inventory methodologies, these results are in line with the hypothesis that the amount of organic matter in the soil is increasing, but the decomposition rate is decreasing. To further study, we analyse *TER* guided by interpreting Equation (1).

**FIGURE 3:**
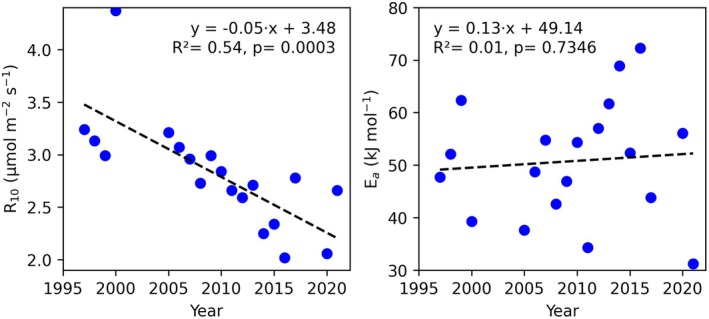
Temporal trends of the fitted R_10_ (left) and E_a_ (right) over the years based on daily mean data from October to March.

Figure 3 shows the temporal variation in the parameters $R_{10}$ and $E_{a}$. The fitted seasonal $E_{a}$ shows interannual variation but no significant long‐term trends over the study period (*r* ^2^ < 0.1 and *p* > 0.73). This implies that the temperature sensitivity of soil respiration did not change systematically during the period of record. However, the fitted seasonal $R_{10}$ exhibits a significant decreasing trend over the study period (*r* ^2^ = 0.54, *p* = 0.0003). Over the 24‐year period of record, this would imply a decrease in TER of 36% (Equation (1)). However, in the same period, the soil temperature has increased by 0.05°C·year^−1^, nearly 1.25°C since 1997. The net effect of decreasing *R*_10_ and increasing *T*_soil_ on *TER* is −29%. This offers additional proof that the soil organic matter has turned more resistant against decomposition over time. In the Discussion, we will discuss ways forward for including a pH‐dependent *R*_*10*_ formulation.

### 3.3 Soil Incubation Experiments

**FIGURE 4:**
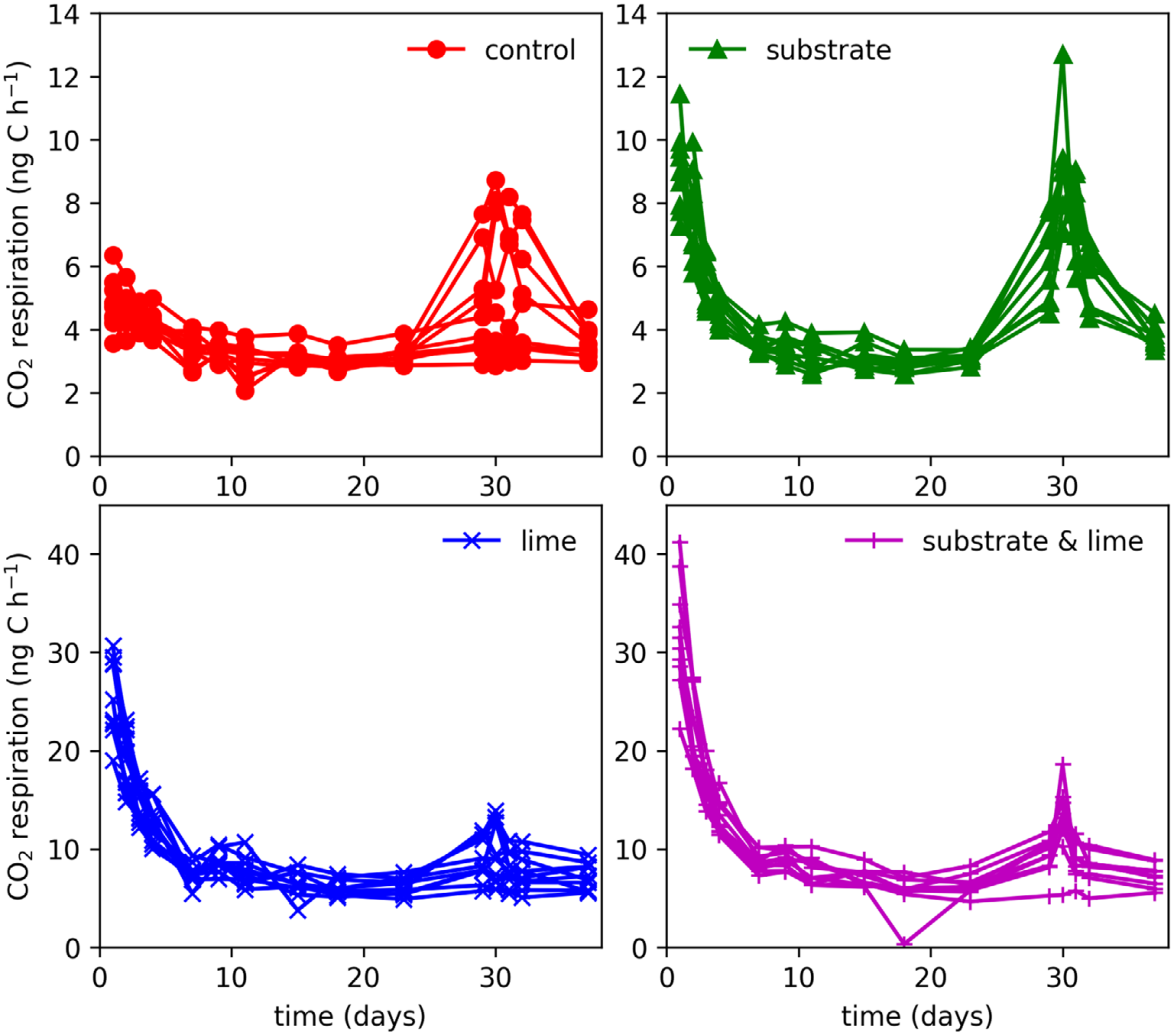
Soil respiration rates for the laboratory‐based soil incubation experiment measured in CO_2_ production in ng C kg^−1^_soil_ h^−1^of dry soil per hour. Top left: Control experiment. Top right: Substrate treatment; bottom left: Lime treatment; bottom right: Substrate + lime treatment; A dose of glucose was added to all laboratory‐based soil incubations of site SP‐I_01 to SP‐I_04 on day 29. Note the different extents of the y‐axes of the top and bottom panels.

The Loobos soils had a C/N ratio of 23.1–28.4, with a total carbon percentage between 21.8% and 42.6% (Table S4). The soil pH was assessed at the start and the end of the incubation. The bottles without lime addition showed a pH range of 2.9–3.0 at the start and 2.9–3.1 at the end of the incubation. The bottles with added lime, to increase the soil pH, showed a pH value of 6.8–7.0 at the start and 7.1–7.2 at the end (Table S5). The laboratory‐based respiration experiment on topsoil samples from Loobos indicates a clear impact of both substrate addition and liming on the microbial respiration rates, as shown in Figure 4. In the bottles with substrate addition, the soils received a dose of glucose at the start of the experiment (*t* = 0 day) and at *t* = 29 days of incubation. In both instances, elevated respiration rates were observed for up to 5 days after addition (Figure 4). In the bottles with lime addition, a similar pattern was observed, with overall elevated CO_2_ respiration rates. It should be stressed that the current experiment did not distinguish between microbial CO_2_ respiration and CO_2_ production due to carbonate dissolution from the lime added. However, for the entire duration of the experiment, the respiration rate of the lime‐treated soils was higher than that of the soils that did not receive a lime treatment. The soils that received the combined treatment with substrate and lime show an accumulated effect (Figure 4).

After 29 days, a dose of glucose was also added to 4 control bottles and 4 lime‐only bottles (SP‐I_01 to SP‐I_04) to investigate whether substrate limitation was persistent throughout the experimental treatment. The results show similar patterns to the soils that previously received glucose, indicating that substrate limitation is persistent in these soils, irrespective of the treatments in this study.

## 4 Discussion

Within FLUXNET, an increasing number of sites now offer data records spanning multiple decades, which may be studied for effects of climatic change and/or environmental disturbances. These long‐term datasets coincide with a period marked by the most rapid and pronounced climate changes in recent millennia, alongside short‐term climatic fluctuations and anthropogenic environmental disturbances (*cf*. Horemans et al. 2020). This presents a timely and compelling opportunity to investigate long‐term trends in ecosystem carbon exchange.

In this context, we present a 26‐year record of total ecosystem respiration (*TER*) from a pine forest in the Netherlands. The data reveal a pronounced downward trend in *TER*, resulting in a reduction of tens of percents between 1997 and 2021. This shift in *TER* implies that eddy covariance‐based estimates of the net ecosystem exchange (*NEE*) at this site are, for a large part, driven by a slowdown in the observed carbon emissions. This raises critical questions: What mechanisms underlie the pronounced *TER* decline observed at Loobos? And are similar processes influencing other FLUXNET sites?

### 4.1 Methodological Aspects

We first examined potential methodological factors related to instrumentation and data processing (Section S1). One notable issue is canopy growth toward the eddy covariance system—an effect likely common across many FLUXNET forest sites. This growth reduces the effective measurement height, potentially altering the eddy size distribution, flux footprint area and the acceptance rate of u_*_ filtering (Munger et al. 2012; Reitz et al. 2022). We show that these effects are real but cannot explain the large negative trend in *TER* observed at our site.

Interestingly, the installation of a second, taller tower with updated instrumentation in 2021 coincides with a marked increase in *TER*—exceeding even the 1997 levels. This apparent trend break aligns temporally with the new tower's deployment. Nevertheless, the inability to attribute the first tower's negative *TER* trend to software, footprint, or u_*_ filtering issues would logically extend to the second tower's data as well. Van der Molen, van der Snellen et al. (2026) report strong agreement between *NEE* measurements from both towers during the brief period of overlap, supporting data consistency across the two platforms. It is worth noting that the winter of 2023–2024 was exceptionally wet, restoring groundwater levels after several years of relative dryness, including the extreme drought of 2018.

When reverting to the non‐gapfilled/partitioned *NEE* data (Appendix S1), we observed frequent negative nighttime *NEE* (negative fluxes imply a downward flow of CO_2_, from atmosphere to vegetation). We found that the majority of these data occurred in periods of rain in the preceding hours. Inspecting the remaining negative nighttime *NEE* shows that it may occur in conditions of advection of CO_2_‐rich air, which is then entrained into the below‐canopy air, either or not associated with intermittent turbulence. Similarly, enhancement of positive nighttime *NEE* may occur in situations with advection of CO_2_‐poor air, which is, however, more difficult to detect. Flagging and removing negative nighttime *NEE* data could thus cause a positive bias in *TER* estimates. Hence, we call for clear and commonly accepted data selection criteria.

### 4.2 Cross‐Site Comparison

A broader analysis across FLUXNET sites with similarly long records indicates that several medium‐aged to mature forest stations also exhibit declining *TER* trends (see Figure 2, Table S2). This finding is striking, as the prevailing ecosystem carbon cycling framework by Odum (1969) posits that carbon content in aging forests should increase until carbon uptake is balanced by ecosystem respiration—implying that *TER* should converge toward *GPP*. A sustained decline in *TER* is therefore unexpected, unless driven by climatic or environmental disturbances, forest harvesting, or widespread tree mortality. Considering that climate extremes occur more frequently now than decades ago, and many FLUXNET sites are located in environmentally polluted and anthropogenically disturbed locations, it is realistic to expect that the health of ecosystem is challenged on a continental scale and that the signals will be detected at FLUXNET sites.

### 4.3 Biogeochemical Processes That Could Explain Declining TER

We next examine biogeochemical processes that could account for the pronounced decline in total ecosystem respiration. Our analysis indicates that soil respiration contributes approximately 69% of *TER*. This is in line with older studies done in other forested ecosystems, which reported contributions 63% (Janssens et al. 2001) and a seasonally variable contribution of 50%–95% (Curiel Yuste et al. 2005). Tang et al. (2014) show evidence that in maturing forest photosynthesis rates decrease faster than the autotrophic respiration rates decrease. Given that at our site the aboveground biomass still increased by 49% between 2000 and 2025, we do not expect a strong decrease in the autotrophic respiration. Hence, we hypothesised that the observed *TER* decline must be driven by a reduction in soil respiration.

Further investigation reveals that the organic layer above the mineral soil holds most of the soil carbon, 67% as measured in 2009. Remarkably, between 2009 and 2024, the mass of this organic layer has doubled, despite a reduction in its physical thickness, suggesting an ongoing accumulation of organic matter. The likely drivers are sustained needle litter input combined with reduced decomposition rates, pointing to a slowdown in microbial activity and carbon turnover within the organic layer. These results are corroborated by model simulations of biomass and soil organic matter turnover in different atmospheric deposition scenarios (Chertov et al. 1999).

We tested the effect of soil temperature on *TER* by fitting an Arrhenius function describing *TER* as a function of soil temperature (Lloyd and Taylor 1994; Jacobs et al. 2007). This analysis revealed a decrease in the reference respiration rate at 10°C (*R*_10_), while temperature sensitivity remained largely unchanged. The 1.25°C increase in soil temperature over the study period was insufficient to offset the declining *R*_10_, indicating that decomposition rates under reference conditions have diminished. This supports the conclusion that soil organic matter is accumulating as decomposition slows; the second process reinforces the first.

To further explore the *TER* trend, we conducted a series of soil incubation experiments that showed substrate and pH limitations (van Groenigen and Zwart 2007). Glucose‐amended samples exhibited a rapid respiration peak lasting approximately 5 days. The magnitude of this response was comparable to the amount of glucose added, suggesting that microbial communities are present but constrained by limited substrate accessibility. Lime‐amended samples also showed elevated respiration, sustained for at least 36 days, indicating that microbial activity is additionally inhibited by low pH conditions.

The extremely low pH of the organic layer (2.9) is likely linked to sustained and substantial atmospheric deposition of ammonia and nitrogen oxides at the site (Melman et al. 2025). According to CLO (2025), nitrogen deposition rates were approximately 38 kg N·ha^−1^·year^−1^ in the 1990s, decreasing to 21 kg N·ha^−1^·year^−1^ by 2023—still representing exceptionally high nitrogen loads. The long‐term effects of sustained acid deposition on the acidity of soil organic matter remain unclear—whether they are immediate or cumulative. Unfortunately, we lack a continuous time series of soil pH measurements to assess this directly. However, Boxman et al. (2008) documented a decline in soil pH from 3.2 in 1990 to 3.1 in 2006 in a comparable Dutch forest, despite a significant reduction in acid deposition over time. This suggests that acidification processes may persist even under declining deposition rates. Horemans et al. (2020) show a recovering pH after historical acid deposition for the FLUXNET site Brasschaat (BE‐Bra), where the pH increased from 3.3 in 1999 to 3.9 in 2014. Low soil pH (2.9–3.5) is a common characteristic of pine forests (Borken et al. 2002). Staszel‐Szlachta et al. (2025) found an average soil pH of 3.98, while Zhang et al. (2025) measured an average soil pH of 6.71, both in forests with the same vegetation as in our study. This is in accordance with other studies, in which low pH in the soil was shown to decrease microbial activity and, consequently, lower respiration (Breugem et al. 2024). Hence, it is clear that the soil pH at our site is still extremely low, regardless of the somewhat decreasing nitrogen deposition loads.

The laboratory‐based soil respiration experiments highlight the combined effects of substrate limitation and acidification on microbial respiration rates in the organic layer above the mineral soil at Loobos (Figure 4). In our liming experiment, soil pH increased by four units, from highly acidic (pH = 2.9) to neutral (pH = 7). While microbial communities are known to adapt to prevailing pH conditions, several studies have shown that microbial growth rates tend to be higher at elevated pH levels (Andersson and Nilsson 2001; Bååth and Arnebrant 1994; Mitsuta et al. 2025). This may explain the consistently higher baseline respiration rates observed after day 6 in lime‐treated soils compared to untreated controls.

However, respiration in the lime‐treated soils did not continue to rise gradually over the course of the 36‐day experiment. This suggests either that low pH is not the only limiting factor for organic matter decomposition at Loobos or that the microbial community was unable to adapt within the experimental timeframe. Supporting evidence from a similar study in Swedish spruce forests showed that liming effects on respiration can emerge within 2–7 days, depending on soil type (Pettersson and Bååth 2003). Likewise, a short‐term experiment on acid black soils under soybean cultivation found that liming enhanced microbial activity, as indicated by fluorescein diacetate assays (Li et al. 2022).

Interestingly, the carbon‐to‐nitrogen (C:N) ratio in the organic layer at Loobos remained unchanged between 2009 and 2025 (Table S2). This is notable, as aging soils typically exhibit a gradual decline in C:N ratio due to microbial biomass accumulation. The absence of such a trend further supports the conclusion that microbial activity is limited in this system. A meta‐analysis by Hao et al. (2021) found supporting evidence that microbial turnover is constrained in acidic forest soils. Additional studies underline the low organic matter turnover and potential nutrient limitation in low pH soils (Liu et al. 2019; Li et al. 2025). This can regulate soil organic matter gains through intensified plant‐microbe competition, as shown for temperate forests (Zhou et al. 2025). This further supports that microbial activity is constrained at Loobos.

At Loobos, we interpret the persistently low pH, the pronounced responses to glucose and lime additions, and the accumulation of organic matter atop the mineral soil as indicators of severely impaired organic matter decomposition. We hypothesise that ongoing nitrogen deposition—though reduced—continues to drive acidification. When combined with annual needle litter input, which contributes recalcitrant organic material, and a reducing diversity of soil microbial community (Rousseau et al. 2024), these conditions increasingly constrain microbial activity and organic matter decomposition.

### 4.4 Decreasing TER in a Larger Perspective

The observed decline in total ecosystem respiration at Loobos accounts for a substantial portion of the net carbon uptake. The decrease of 27% in the *TER* of 1350 gC·m^−2^·year^−1^ in 1997 (Table S1) corresponds to a prevented emission of 335 gC·m^−2^·year^−1^ by 2021, i.e., 70% of the annual *NEE* of about 500 gC·m^−2^·year^−1^ (Zhao et al. 2026). This raises a critical question: How sustainable is this carbon sink in the long term, given the evident suppression of soil biological activity?

Why is this relevant for the FLUXNET and carbon cycle research community? To the best of our knowledge, this is the first time that a decline in TER in relation to sustained nitrogen deposition is observed using long‐term FLUXNET data. Although *TER* is not the most commonly studied variable within FLUXNET, receiving less attention than *GPP* and *NEE*, it deserves to be studied. With the growing availability of long‐term FLUXNET datasets, we are now able to investigate trends at decadal scales. Understanding these trends and attributing fluxes to changes in carbon stocks is urgently needed (Randerson et al. 2025).

Our findings show that a negative trend in *TER*, though counterintuitive, is not unique among the 24 FLUXNET sites with the longest continuous records (Section S2). Such declines imply a contribution to a net atmospheric carbon sink, which could be interpreted as a climate‐positive signal. However, decreasing *TER* is unexpected in aging forests and may point to climatic or environmental disturbances. The increasing frequency of droughts, heatwaves, floods, biotic stressors such as pests and diseases and anthropogenically induced pollution may push ecosystems toward thresholds of resilience (Anderegg et al. 2022). In this context, Loobos may serve as an early warning for other FLUXNET sites experiencing environmental stress.

We propose an integrated approach that combines observation, data analysis and modelling. From an observational standpoint, the increasing length of data records calls for a reassessment of methodological impacts related to forest growth, stand density and shifts in ecosystem composition. Disturbance indicators should be systematically monitored and documented at FLUXNET sites. Routine pH measurements as straightforward and valuable observations should become standard. ICOS conform tree and soil inventories can help detect changes in species composition, tree vitality and soil organic matter, as well as soil microbial composition. To deepen our understanding, we recommend incorporating measurements of deposition fluxes of reactive nitrogen compounds and other key pollutants or signal species (e.g., ozone, VOCs), as well as lateral transport of carbon and nitrogen. In regions affected by acidification, we advocate for establishing links between acid deposition, pH levels, microbial community dynamics and organic matter accumulation, e.g., by quantifying microbial carbon use efficiency (Tao et al. 2023). We anticipate that lag times may exist between nitrogen deposition, acidification of soil organic matter and changes in microbial community, because we observe a continuous decline in *TER* while nitrogen deposition decreased. Such continuous research should result in improved pH‐dependent respiration rate parameterisations.

From a data analysis perspective, FLUXNET datasets should be systematically examined for trends in net ecosystem exchange, and further partitioned into gross primary production and total ecosystem respiration. Persistent carbon sinks or sources (Janssens et al. 2001) must be attributed to changes in above‐ground (Randerson et al. 2025) and soil carbon stocks, supported by tree and soil inventories and potentially enhanced by lidar and remote sensing technologies.

On the modelling front, ecosystem models should incorporate a functional response of soil organic matter to nitrogen availability, pH variations and microbial community composition (Chertov et al. 1999; Chen et al. 2016; Breugem et al. 2024; Rousseau et al. 2024; Zhang et al. 2025). Dynamic vegetation models—including those used in atmospheric inversions—must be validated against long‐term *NEE*, *GPP* and *TER* data from FLUXNET. We encourage close collaboration between site investigators and modellers, as deep expertise in both site‐specific conditions and model frameworks is essential to meet this challenge.

## 5 Conclusion

We study a pronounced negative trend in total ecosystem respiration at our FLUXNET site Loobos, with respiration levels decreasing by tens of percents between 1997 and 2021. While we investigated several methodological factors that might explain this decline, none were sufficient to account for the magnitude of the observed trend. However, we do acknowledge an unexplained increase in TER following the installation of a second, taller tower.

We find that the soil organic matter mass per unit area has doubled over the study period. This trend persists regardless of soil temperature changes or variability, providing further evidence that total ecosystem respiration (*TER*) is constrained by the rate of decomposition rather than climate variations or the availability of organic matter. Soil incubation experiments support the idea that current respiration rates are limited by substrate quality and acidity. The addition of glucose, a readily accessible substrate, revealed that soils at the Loobos site host an active microbiome, but its activity is inhibited by the site's strongly acidic conditions (pH = 2.9). We attribute this acidity to sustained and elevated nitrogen deposition at the site. While methodological explanations for the declining carbon sink remain possible, our results provide strong evidence for a biogeochemical explanation.

The long‐term record at our FLUXNET site Loobos has enabled us to detect a clear downward trend in *TER*. We argue that this decline contributes substantially to the net atmospheric carbon sink observed at the site. However, this raises important questions about ecosystem health and whether the observed sink is sustainable in the long term.

Loobos is subject to high nitrogen deposition and as such serves as a warning signal for the impacts of environmental disturbances on ecosystem functioning, stability and sustainability. However, similar anthropogenic and climate‐induced disturbances are likely affecting other FLUXNET sites as well, and indeed, negative trends in total ecosystem respiration (*TER*) have been observed at several long‐term sites.

For global carbon cycle research, including experimental studies, forward ecosystem and climate modelling, and atmospheric inverse modelling, it is essential that observed trends in carbon fluxes are properly attributed and explained. Changes in carbon and nitrogen fluxes should be supported by corresponding routine stock measurements. To advance our understanding of the carbon cycle, we must continuously refine models through confrontation with observational data, improving the representation of processes such as the potential inclusion of soil acidification dependence in ecosystem respiration representations.

## Author Contributions

**Michiel K. van der Molen:** conceptualization, formal analysis, investigation, methodology, supervision, visualization, writing – original draft. **Marnix van de Sande:** formal analysis, investigation, writing – original draft. **Michiel in 't Zandt:** conceptualization, formal analysis, investigation, methodology, validation, writing – original draft. **Tori Saccomandi:** formal analysis, investigation, methodology, writing – original draft. **Sophie L. Baartman:** formal analysis, investigation, methodology, supervision, writing – original draft. **Hong Zhao:** data curation, formal analysis, investigation, methodology, writing – original draft. **Jordi Vilà‐Guerau de Arellano:** conceptualization, methodology, supervision, writing – original draft.

## Funding

The first tower Loobos measurements between 1997 and 2021 were made possible through the grants below:

- The ‘CarboEurope‐Integrated project’ supported by the European Commission through contract GOCE‐CT2003‐505572.
- The ‘EUROFLUX project (ENV‐CT95‐0078)’ funded by the European Union Fourth Framework Programme.
- The ‘Infrastructure for Measurements of the European Carbon Cycle (IMECC) project’ funded by the European Union (Framework Program 6).
- The ‘GHG‐Europe project’ funded by the European Union (Framework Program 7)
- The ‘Hydrology and water balance of forest in the Netherlands project’ funded by the Dutch Ministry of Agriculture, Fisheries and Nature Management, the Dutch Forestry Commission (SBB).
- The ‘Integrated observations and modelling of greenhouse gas budgets at the ecosystem level in the Netherlands project (ME1)’ supported by the Dutch National Research Program Climate Changes Spatial Planning.
- The ‘Climate Research Program on Climate Change of Wageningen University and Research’ supported by the Ministry of Agriculture, Nature and Food Safety of the Netherlands.
- The Ruisdael Observatory funded by the Dutch Research Council (NWO) through a National Roadmap for Large‐Scale Research Facilities.

The second Loobos tower and its instrumentation from 2021 onward is funded by the Dutch Science Fund (NWO, 2025) via the Ruisdael Observatory (2025) grant. The station is built on land owned by the Dutch National Forest Service (Staatsbosbeheer). We acknowledge payment of the ICOS station fees by the Ministry of Infrastructure and Water Management.

All sources of institutional, private and corporate financial support for the work within the manuscript must be fully acknowledged, and any potential conflicts of interest noted. If in doubt, please check the Open Funder Registry for the correct nomenclature: https://www.crossref.org/services/funder‐registry/.

## Conflicts of Interest

The authors declare no conflicts of interest.

## Supporting information


**Data S1:** gcb70849‐sup‐0001‐Supinfo.pdf.

## Data Availability

The data that support the findings of this study are openly available in https://doi.org/10.5281/zenodo.15721310 (Zhao 2025), van der Molen et al. (2026a, 2026b, 2026c, 2026d, 2026e) and van der Molen, van de Sande, et al. 2026. The FLUXNET data for the Loobos second tower and the other FLUXNET sites are available via the ICOS carbon portal (https://www.icos‐cp.eu), see also Table S2 for the specific references, listed in the Supporting Information. The Python processing scripts are archived at https://git.wur.nl/molen050/loobos‐declining‐ecosystem‐respiration‐gcb.
