# Supplementary material for: Declining Ecosystem Respiration Linked to Nitrogen Deposition: Insights From a 26‐Year FLUXNET Record
Source: Glob Chang Biol. 2026 Apr 13;32(4):e70849. doi: 10.1111/gcb.70849 (PMC13071790; doi:10.1111/gcb.70849)
Supplement: Supplementary file 1 — Data S1: gcb70849‐sup‐0001‐Supinfo.pdf. [file GCB-32-e70849-s001.pdf]

Supporting Information

S1 | *TER* trend and sensitivity

Eddy covariance measurements of carbon dioxide flux are collected at Loobos since 1997 in combination with profile measurements of CO_2 concentration. The measurements in the first tower at 27 m height until 2023 are described in detail in Zhao et al. (2026) and the ones starting in 2021 at 38.2 m height in a second tower are described in detail in van der Molen et al. (2026). The raw data were processed into half hourly net ecosystem exchange (NEE) fluxes using the AltEddy software package (Elbers et al., 2011, Mauder, 2008), including all customary operations and corrections (*c.f.* wind rotations, Webb and Schotanus corrections, frequency attenuation corrections, the 'Burba' correction for sensible heat generation along the open path gas analyser, Webb et al., 1980, Schotanus et al., 1983, Moore , 1986, Burba et al., 2006, 2008). The half hourly fluxes were partitioned into gross primary productivity (*GPP*) and total ecosystem respiration (*TER*) based on nighttime TER following Reichstein et al. (2005). Using monthly mean *TER*, we find a respiration rate of 113.1 ± 5.8 gC m^{-2} $month^{-1}$ in 1997 with a downward trend of 1.16 ± 0.41 gC m^{-2} $month^{-1}$ yr^{-1} reducing *TER* to about 80 gC m^{-2} $month^{-1}$ in 2021, with p = 0.0005 (Figure 1, table S.1).

The measurements collected in the second tower were submitted to the ICOS carbon portal (https://ICOS-cp.eu, van der Molen et al., 2026a-e). The ICOS Ecosystem Thematic Centre processes the raw data to half hourly partitioned fluxes. Here we use the half hourly FLUXNET product (https://meta.icos-cp.eu/resources/cpmeta/etcL2Fluxnet), because it was made available starting January 2023, earlier than the official labelling date (10 May 2023). Surprisingly, the monthly *TER* ('*RECO_NT_VUT_MEAN*') were higher again and more variable with an average and standard deviation of 143.7 ± 61.2 gC m^{-2} $month^{-1}$ from January 2023 to December 2024.

The jump in *TER* in 2023 is at the time of changing instrumentation, measurement height (footprint, eddy size distribution) and data processing from the first tower dataset to the second tower's. It seems likely that the jump in *TER* is related to this change. However, van der Molen et al. (2026) show good agreement in *NEE* observations during a period of overlapping data, particularly for negative (daytime) *NEE*, however. With the still short period of record of the second tower and the large variability in *TER* it is currently not feasible to detect if the downward trend continues after 2021. However, the sudden jump was interpreted as an invitation to investigate whether measurement errors could have been the cause of the trend detected in the first tower's *TER*.

At Loobos, the trees were 15.3 m tall on average in 1996 and since then grew about 0.16 m yr^{-1} in height to 20.5 m in 2025 (Zhao et al., 2026). Hence, the trees grew towards the eddy covariance system, which was installed at a height of 27 m until 2021.

The effective measurement height (*z-d*) decreased from 16.8 m in 1996 to 14.3 m in 2021, where we assume the displacement height *d* as 2/3 of the tree height. This may have affected the observed *NEE* in three ways.

1) The eddy size distribution shifts to smaller eddies (higher frequencies). This does affect the high frequency corrections for path length averaging, sensor separation, input line averaging, density fluctuations, etc. as well as low frequency corrections for coordinate rotation and averaging length. However, corrections for these effects were implemented in the data processing (*cf.* Moore , 1986, Webb et al, 1980, Foken et al., 2004) and the software was successfully cross validated with other software packages (Mauder et al., 2008). Flux calculations with EddyPro show that the spectral correction factor for CO_2 flux ($0.3 < u^* < 1.0$ m s^{-1}) is below 1.02 when the system was installed at 27 m, (z-d) = 13.5 m and below 1.04 when the system was installed at 38.2 m (z-d) = 24.7 m, reflecting

large eddy sizes well above a rough surface. Canopy height is one of the input parameters to AltEddy the software package and was updated regularly. With a relatively small change in observation height $\Delta(z\text{-}d) = 2.5$ m and the effects accounted for in the processing software, we consider it unlikely that the change in eddy size distribution can explain the decrease of tens of percents in *TER* in 24 years.

2) With the decrease in effective measurement height, the size of the eddy covariance footprint decreases. An analysis of the 80% fetch data (van der Molen et al., 2026) shows that the footprint decreased from 1691 m between 1997 and 2000) to 936 m between 2018 and 2021 in nighttime conditions with a $u_* > 0.3$ m s^{-1}. While this represents a significant change in footprint area, an analysis of the *TER* as a function of the 80% fetch distances for both the short period 1997-2000 and the entire period of record show that the observed total ecosystem respiration rates are independent of the footprint size (slope = -0.044 µmol C m^{-2} s^{-1} $km^{-1}{}_{footprint}$, with a mean *TER* = 2.8 µmol C m^{-2} s^{-1}, p=0.61). This result is consistent with the homogeneity of the forest composition, aboveground biomass up to 1-3 kilometres around the tower (van der Molen et al., 2026). In 2024 a soil inventory was performed according to ICOS instructions. The results are not available yet, but while doing the soil sampling, our impression was that the soil and organic layer are rather uniform. Concludingly, the evidence we have does not suggest that changes in the footprint area during the period of record may explain the large observed decrease in *TER*.

3) Dolman et al. (2002) propose a u_* threshold value of 0.25 m s^{-1}, below which turbulence intensity was insufficient for efficient mixing of respired carbon dioxide out of the below-canopy air. This analysis was based on the 1997 eddy covariance data. Because u_* generally decreases with height from the canopy top upwards (Santana et al., 2018), the eddy covariance system may have been exposed to progressively more intense turbulence. Indeed, we see an increase of 1.4 % to 13 % in seasonally mean u_* in most seasons, except spring, over the period 1997-2021. This may be caused by a superimposition of the growing trees and changing wind climatology (speed and direction).

Using a constant u_* threshold value to filter out non-turbulent periods, results in a decrease of the relative fraction of rejected data from 38 % in 1997 to 32 % in 2020 (Fig.S.1a,b). When working with a constant u_* threshold value, the selection criterion would thus become progressively more strict, which could have an effect on the resulting estimate of TER.

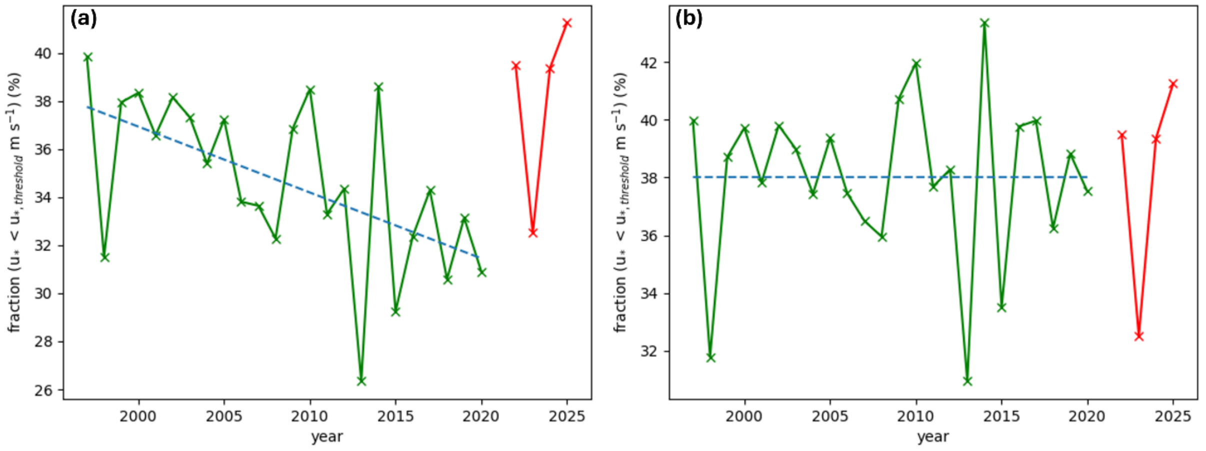

Figure S.1. (a): Green line: Fraction of eddy covariance data with $u_* < 0.3$ m s^{-1} as a function of time into the period of record. The blue dashed regression line has a slope of -0.2741 yr^{-1}. (b), green line: Fraction of eddy covariance data with $u_* < u_{*,threshold}$ (Eq. S1). The regression line has a slope of 0.0000 yr^{-1}. The green data points are measured in the first tower, the red ones in the second tower. Both left and right, the red line indicates the fraction of eddy covariance data with $u^* < 0.33$ m s^{-1}.

To test the impact of removing the implicit relaxation of the u_* threshold criterion, we empirically developed a time dependent u_* threshold value ($u_{*,threshold}$):

$$u_{*,threshold} = b + a \times t \qquad \text{(Eq. S.1)}$$

where t is the decimal year minus 1997 (t = 0 yr on 1997-01-01 and t = 28 yr on 2025-01-01) and b is the threshold value at t = 0 yr. We perform three sensitivity analyses:

i) a = 0.000 mm s^{-1} yr^{-1} and b = 0.25 m s^{-1},
ii) a = 0.000 mm s^{-1} yr^{-1} and b = 0.30 m s^{-1}, and
iii) a = 2.323 mm s^{-1} yr^{-1} and b = 0.30 m s^{-1}.

The value b = 0.25 m s^{-1} is according to Dolman et al. (2002) and the value b = 0.30 m s^{-1} is somewhat higher than that value to be absolutely sure we select turbulent conditions. With the coefficient a = 0.002323 m s^{-1} yr^{-1} in iii) the trend in the rejected fraction of -0.2741 yr^{-1} (Fig. S.1 left) becomes 0.0000 yr^{-1} and is effectively removed (Fig. S.1 right).

Next we quantified the sensitivity of the derived trend in *TER*. Here we estimate *TER* as the mean *NEE* (van der Molen et al., 2026f) with a u_* larger than the threshold value and in dark conditions (from an hour after sunset to an hour before sunrise). Interestingly, this resulted in a dataset with quite frequent negative nighttime *NEE*. The majority of these negative NEE occur in conditions of precitation in the previous 2 hours or in low wind speed conditions (u < 1 m s^{-1}) or when the quality flags indicated low quality flux data. Removing data in these conditions removed the majority of the negative nighttime *NEE* data, including the most negative ones. The negative nighttime *NEE* data remaining were sometimes associated with increasing above canopy CO_2 concentrations and/or intermittent turbulence, suggesting the occurrence of entrainment of CO_2 rich air into the canopy layer. We group the monthly data over the hours of the day and take the average over the mean diurnal cycle. This is a robust method, which makes the results relatively unsensitive to data gaps, missing profile data and model fits. It may however result in somewhat lower *TER* values, because the daytime *TER* is not estimated, like in the FLUXNET estimates. For the analysis of the sensitivity of the trend, we consider this acceptable.

Table S.1 shows that the choice of a constant or variable u_* threshold value has some influence on the magnitude of trend in nighttime *NEE*. Except in winter, the relative trends remain of similar magnitude. In winter, the estimated trend is a lot larger. We attribute this to the small winter-time nighttime *NEE* and the frequent occurrence of long periods of precipitation. However, in all sensitivity scenarios and all seasons, the negative trend is robust and in the order of several tens of percents over the 26 years.

Table S.1. The trend in TER and nighttime NEE with a constant (0.25 and 0.30 m s^{-1}) and variable (Eq. S1) $u_{*,threshold}$. In brackets is the relative change over the period of record (26 years × slope / intercept) and the p-value. Trends printed in bold are statistically significant at the 5% level.

Season	***TER* trend (FLUXNET product)**	**Nighttime *NEE* trend i) ($u_{*,threshold}$ = 0.25 m s^{-1})**	**Nighttime *NEE* trend ii) ($u_{*,threshold}$ = 0.30 m s^{-1})**	**Nighttime *NEE* trend iii) (variable $u_{*,threshold}$)**
	gC m^{-2} $month^{-1}$ yr^{-1}	gC m^{-2} $month^{-1}$ yr^{-1}	gC m^{-2} $month^{-1}$ yr^{-1}	gC m^{-2} $month^{-1}$ yr^{-1}
**DJF**	**-1.14 (-54%, p=0.00)**	**-1.55 (-90%, p=0.00)**	**-1.66 (-94%, p=0.00)**	**-1.64 (-92%, p=0.00)**
**MAM**	**-1.36 (-32%, p=0.01)**	**-0.98 (-34%, p=0.02)**	**-0.89 (-32%, p=0.04)**	**-0.88 (-31%, p=0.05)**
**JJA**	**-1.29 (-19%, p=0.00)**	**-1.63 (-29%, p=0.01)**	**-1.47 (-26%, p=0.00)**	**-1.58 (-27%, p=0.00)**
**SON**	**-1.21 (-27%, p=0.03)**	-0.54 (-16%, p=0.53)	-0.56 (-17%, p=0.53)	-0.67 (-20%, p=0.46)
**ALL**	**-1.16 (-27%, p=0.00)**	-0.95 (-29%, p=0.12)	-0.92 (-28%, p=0.14)	-0.96 (-29%, p=0.12)

Based on the robust, statistically significant trends we find in nearly all scenarios, we conclude that we cannot find an instrumental or data processing explanation for explaining the trends in *TER* between 1997 and 2021. We observe that the trend in nighttime *NEE* appears to be different than the trend observed in *TER*, and assume this is because of the gap filling and flux partitioning underlying the FLUXNET *TER* product. The nighttime *NEE* product is only based on direct eddy covariance measurements.

The period of record of the second tower's data (2023-2024) is still only 2 years long, and the variability in monthly *TER* and in the fraction of data with u_* below the threshold value is large, which makes it difficult to define a u_* threshold value for the second tower data. The second tower data show a larger *TER* than at the end of the first tower's period of record. We do not know yet how to attribute this, also considering the points discussed above. We therefore omit the data from 2021 until we have a longer period of record of the second tower.

S2 | Comparison of *TER* trend with other sites

The FLUXNET, AMERIFLUX and ICOS-ETC datasets contain numerous long-term records of *TER* measurements. To compare our findings for the Loobos flux measurement site to other forested measurement sites, we calculated the trends in *TER* at other flux sites. We included sites that were measuring *NEE* in temperate or boreal forests for at least 5 consecutive years, and that included processed *TER* in their data records based on nighttime flux separation (Reichstein et al., 2005). This resulted in a list of 23 sites, which can be found in table S.2. After noticing some artifacts in *TER* fluxes that had been computed during periods where local temperature was gap-filled with ERA5 reanalysis data, we decided to filter out all data containing ERA5 temperature data and removed years (Table S.2) and seasons (Fig. 2) with less than 50% data remaining. Data was aggregated by taking a yearly mean for table S.2 and a seasonal mean for Fig. 2 in the main text, and alltrends were fitted using linear least-squares regression.

Table S.2: Ecosystem flux measurement sites included in the cross-comparison. In the column PFT (Plant Functional Type), DBF indicates 'Deciduous Broadleaf Forest', ENF indicates 'Evergreen Needleleaf Forest', and MF indicates 'mixed forest'.Slopes with a p-value better than 0.05 are printed in bold.

PFT	Site code	Site name	Years	Slope	p	Citation
DBF	DE-Hai	Hainich	2000-2012	-0.68	0.09	Knohl et al., 2016a
DBF	DE-HoH	Hohes Holz	2019-2024	-5.37	0.22	Rebmann et al., 2025
DBF	DE-Lnf	Leinefelde	2002-2012	-1.66	0.16	Knohl et al., 2016a
DBF	DK-Sor	Soroe	1996-2014	0.59	0.37	Ibrom and Pilegaard, 2016
DBF	FR-Fon	Fontainebleau-Barbeau	2005-2014	0.87	0.07	Berveiller et al., 2016, 2025
DBF	IT-Col	Collelongo	1996-2014	**1.11**	**0.01**	Matteucci, 2016
DBF	US-Ha1	Harvard Forest	1991-2012	**1.74**	**0.03**	Munger, 2016
DBF	US-MMS	Morgan Monroe State Forest	1999-2014	-0.56	0.10	Novick and Phillips, 2016
DBF	US-UMB	Univ. of Mich. Biological Station	2000-2014	0.43	0.35	Gough et al., 2016
ENF	CA-Qfo	Quebec – Eastern Boreal, Mature Black Spruce	2003-2010	0.75	0.42	Margolis, 2016
ENF	CH-Dav	Davos	2019-2024	8.78	0.09	Feigenwinter et al., 2025
ENF	DE-RuW	Wustebach	2012-2024	**6.17**	**0.00**	Schmidt et al., 2025
ENF	FI-Hyy	Hyytiala	1996-2014	0.18	0.57	Mammarella et al., 2016
ENF	FI-Let	Lettosuo	2017-2024	-0.31	0.90	Korkiakoski et al., 2025
ENF	FI-Var	Varrio	2017-2024	1.27	0.10	Kolari et al., 2025
ENF	FR-Bil	Bilos	2019-2024	-0.33	0.94	Domec et al., 2025
ENF	RU-Fyo	Fyodorovskoye	1998-2014	**-2.50**	**0.01**	Varlagin et al., 2016
ENF	SE-Htm	Hyltemossa	2018-2024	4.03	0.51	Heliasz et al., 2025
ENF	SE-Nor	Norunda	2018-2024	-2.17	0.91	Kljun et al., 2025
ENF	US-Blo	Blodgett Forest	1997-2006	2.67	0.61	Goldstein, 2016
ENF	US-Ho1	Howland Forest	1996-2023	-0.13	0.78	Ouimette, 2024
MF	BE-Vie	Vielsalm	1996-2014	2.44	**0.00**	Ligne et al., 2016
MF	CH-Lae	Laegern	2004-2014	**-3.71**	**0.05**	Hörtnagl et al., 2025

Fig. 2 in the main text shows the seasonal mean *TER* at these flux sites, together with fitted linear trends. There is a diversity in the sign and magnitude of the trend in *TER*, as documented in Table S.2. Interestingly, while many sites show an upwards trend in *TER*, a small number of sites show no trend in *TER* or suggest that this trend could be negative. Specifically, sites Hainich (DE-Hai, slope = –0.68, p = 0.09), Leinefelde (DE-Lnf, slope = -1.66, p = 0.16), Fyodorovskoye (RU-Fyo, slope = --2.50, p = 0.01) and CH_Lae (slope = -3.71, p = 0.05) hint at a negative trend over time, although for the case of Hainich and Leinefelde this is not shown with statistical significance ($p < 0.05$). Overall, the differing trends in *TER* observed at these various flux sites calls for a more elaborate investigation of local environmental pressures at these sites.

S3 | Relative contribution of soil respiration

Total ecosystem respiration is the sum of soil respiration and above ground biomass respiration. In order to know if the trends in *TER* are caused by changes in soil or above ground biomass respiration, Fig.S.2 shows the comparison of *TER* and soil respiration as measured between 2001 and 2011. Here soil respiration is measured with chamber measurements along a transect of 22 fixed collars (see Zhao et al. (2026) for details and Zhao et al., 2025 and van der Molen et al., 2026f for the data), and thus represents the sum of heterotrophic and root respiration. Overall, the two correlate well (r^2=0.45), and the soil respiration is on average 69% of *TER*. This clearly indicates that the soil respiration contributes the largest part to *TER*. The large trend in *TER* over the 24 year period of record cannot be attributed to above ground biomass respiration alone, because i) the above ground biomass is still growing and ii) even a 100% decrease in above ground biomass respiration would not be enough to equal the trend in *TER*. Consequently, we seek to explain the trend in *TER* primarily by changes in soil respiration, particularly heterotrophic respiration of litter and soil organic matter. With the increase if *GPP* (section 3.1 in the main text), we do not expect a decrease in root respiration and, consequently, we seek to explain the trend in *TER* primarily by changes in heterotrophic respiration.

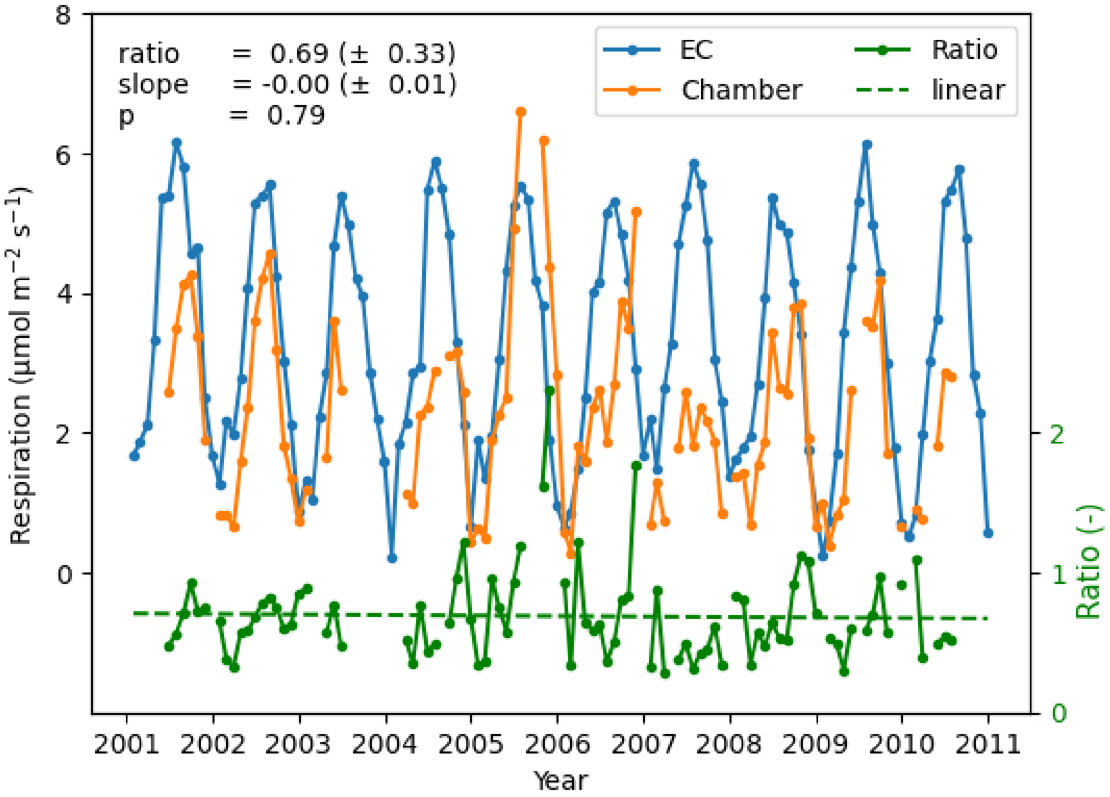

Figure S.2. Comparison of ecosystem respiration measured by the EC tower to the chamber measurement of soil respiration.Figure S.2. Comparison of chamber measurements (labelled 'Chamber') to eddy covariance based TER estimates (labelled 'EC'). Both 'Chamber' and 'EC' are plotted along the left y-axis and represent monthly mean values. The ratio of the two is plotted on the right y-axis (labelled 'Ratio'). The linear regression line is also shown (labelled ('linear'). The mean and standard deviation of the ratio, the slope and standard error of the slope and the p-value of the slope are shown in the top left of the figure.

S4 | Organic layer thickness

In 2009 and 2024 soil inventories were performed in Loobos (Zhao et al., 2025, 2026, van der Molen et al., 2026f). In 2009, the soil was sampled in a raster of 5 × 5 locations with a distance of 75 m in between the locations. The raster was centred around the first tower. In 2024, a soil inventory was performed according to ICOS ETC instructions (https://www.icos-etc.eu/icos/documents/instructions), implying taking 100 soil cores, 5 at 20 randomly assigned first order Sampling Plots (SP-I) in the forest (See Fig. 1 in the main text).

The 2009 inventory shows that 4.7 kg C m^{-2} (67%) of the total soil organic carbon (6.9 kg C m^{-2}) is located in the organic layer on top of the mineral soil. The remaining 33% is distributed over the mineral layer until 100 cm deep. The 100 samples from the 2024 soil inventory have been dried, weighted, but the chemical analyses are not yet available.

Table S.3, interestingly, suggests that the thickness of the organic layer has decreased from 12.6 cm in 2009 to 7.3 cm in 2024, while the bulk density has increased from 0.05 g cm^{-3} to 0.17 g cm^{-3}. As a result, the mass of the organic layer per unit area has doubled. The organic layer mass is comparable with those reported in Borken et al. (2002). There are no indications that the C:N ratio has changed, although the C and N concentrations from the 2024 inventory are not known yet.

These results should be interpreted with care, because the measurement locations, sampling protocols and analysis methods differed between the two inventories and because the standard deviations around the 2009 and 2024 mass per area are large relative to the change.

These results suggest that the soil organic layer has compacted. The organic layer has gained weight and lost air space.

Table S.3. Thickness, bulk density, mass and C:N ratio of the organic layer on top of the mineral soil resulting from soil inventories in 2009 and 2024. (*) The C:N ratio in 2024 is based on the lab experiments (Section 3.3 in the main text, Table S.5).

Year	N	Thickness (cm)	Bulk density (g cm^{-3})	Mass / area (g cm^{-2})	C:N
**2009**	25	12.6 ± 4.1	0.05 ± 0.02	0.61 ± 0.31	25.06
**2024**	100	7.3 ± 3.7	0.17 ± 0.09	1.23 ± 0.88	23.1-28.4 (*)

S5 | Soil incubation experiments

Ten locations were selected for the collection of soil organic matter. They are a subselection of the ICOS first order Sampling Plots (SP-I_NN), where ICOS conform tree and soil inventories are performed (NN refers to the number of the sampling plot, ranging from 1 to 20. They were selected based on the proximity to the eddy covariance tower, and were located within a 760 m radius from the tower (Fig. S.3, Table S.4). The area between 200 and 900 m around the tower contributes 50% and 90% to the flux footprint area of the eddy covariance measurements at night (van der Molen, 2026).

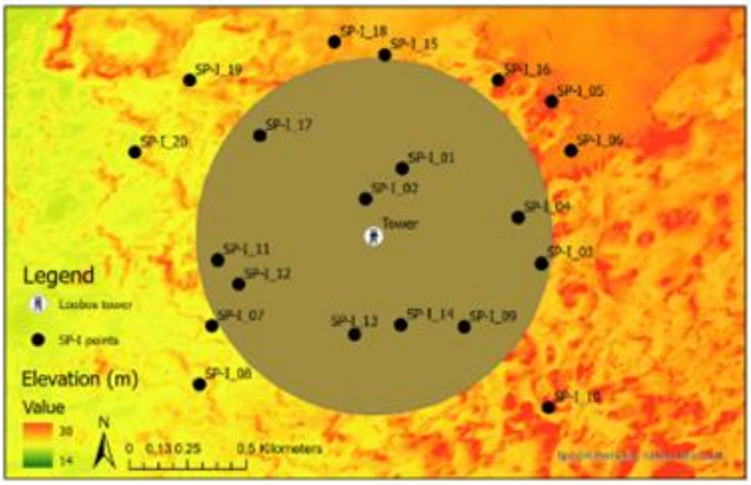

Figure S.3. Location of the 10 sample locations and their proximity to the eddy covariance tower ("Tower") at the Loobos site. The brown circle has a radios of 760 meters, indicating the 10 locations closest to the tower. Graph made on ArcGIS Pro.

Table S.4 shows the geographic coordinates of the sampled locations at Loobos.

Moisture content and water-holding capacity - Per location 105g of sieved soil was oven-dried at 105°C for 24 hours in a forced-air drying oven. If after 24 hours the sample was not dry to the touch, the sample was oven-dried at 105°C for another 24 hours. After oven-drying, the weight was determined to calculate the moisture content of each sample.
For the respiration experiment, we standardized the moisture content at 60% of the water holding capacity (WHC) of each soil. Six grams of the 105°C dried soil was placed on a dry plastic bowl. Approximately 1 ml (+/- 0.1ml) of water was mixed into the soil per addition step. Additions were repeated until the water was visibly separating from the soil.

Table S.4: Geographic coordinates of sampled locations.

Name	LAT (deg N)	LON (deg E)
**SP-I_01**	52.16919	5.74535
**SP-I_02**	52.16802	5.74305
**SP-I_03**	52.16552	5.75398
**SP-I_04**	52.16728	5.75254
**SP-I_09**	52.16311	5.74912
**SP-I_11**	52.16571	5.73377
**SP-I_12**	52.16480	5.73507
**SP-I_13**	52.16284	5.74231
**SP-I_14**	52.16320	5.74521
**SP-I_17**	52.17048	5.73645

For this soil type in particular, the water holding capacity was unexpectedly high, with the soil being able to hold on average double its own weight in water. Therefore, the soil was pressed down with a spoon, and, once water was visibly leaking from the soil, this was considered as an oversaturated soil. The actual WHC for the sample was considered as the average between the last and the second to the last water addition.
Next, the 60% WHC points were calculated for each sample. Eight samples were below 60% WHC. Only two samples were above 60% WHC: SP-I_11 (at 69% WHC) and SP-I_13 (at 71% WHC). These samples were left in an uncovered tray to dry at room temperature on a table for 16 hours. Afterwards, the WHC measurements were repeated. Sample SP-I_11 was at 61% WHC and SP-I_13 was at 64% WHC at the start of the experiment. For all other samples, demineralized water was added to achieve 60% WHC. The associated data can be found in van der Molen et al., 2026f).

Substrate additions and sample incubation – For all bottles, fresh soil equivalent to 50g of dry soil was added. For each location, the amount of water needed for achieving WHC of 60% was added, as described in the previous paragraph. For the glucose addition, 1 ml of a 150 g/l glucose solution in demineralized water was used, achieving a dose of 3 g glucose per kg dry soil. To compensate for the 1 ml volume of glucose addition, 1 ml less demi water was added to control and lime test bottles. For the lime addition we used calcium carbonate and a total of 20% w/w lime per dry soil weight equivalent was added. Once all additions were added, all samples were hand mixed for 30 seconds in a plastic bowl, then added to the experiment bottle. All bottles were closed with cotton wool to allow for gas exchange prior to the respiration incubations. All bottles were incubated in a dark room at 20°C.

All bottles were weighed at the start of every week and the difference with its initial weight was corrected by the addition of demineralized water. This ensured a constant 60% WHC during the experiment.

At day 29 a second dose of 1 ml glucose solution was added to all glucose amended bottles. In addition, 1 ml of the glucose solution was added to the control and lime-only incubations from site SP-I-01 to SP-I_04 to investigate the impact of glucose amendment on unamended samples after incubation.
Gas calculations - At the start of the incubation, the cotton wool was removed and all bottles were ventilated with compressed air for 10 minutes. During ventilation, bottles were gently hand-shaken to detach solid particles from the glass wall. After 10 minutes, bottles were closed with a screw cap with a butyl rubber top. Bottles were incubated for 1 hour in week 1 and for 1.5 hours for the rest of the experiment.

All CO_2 measurements were performed on an INNOVA1412 Photoacoustic Field Gas Monitor (LumaSense Technologies, USA). Compressed air was measured as reference between each sample. During each measurement a total volume of 71 mL is removed from each bottle and replaced with compressed air by the machine. After measuring, the metal caps were removed and replaced with cotton wool. All bottles were incubated in a dark room at 20°C until the next measurement.

At the end of the incubation the headspace of each bottle was determined. All bottles were weighed and filled with water. Bottles were closed with the metal cap and hand shaken for 10 seconds to allow for water to penetrate the soil. A final dose of water added with a squeeze-bottle until the formation of a meniscus. The end weight was used to calculate the total headspace volume of each bottle.

pH and C:N ratio - The pH was measured with a 0.01M CaCl2 extraction before and after the experiment. At the start of the experiment, 5 ml of soil sample was taken after mixing in water, glucose and lime, as described above. At the end of the experiment 5 ml of soil was removed from each incubation bottle. Next, 25 mL of 0.01M CaCl2 solution was added to achieve a 1:5 soil to liquid ratio. Samples were shaken at 120 Hz for 1 hour and then allowed to settle for 1 hour. The pH was measured directly in the liquid phase.

For the C:N ratio a 40 g sample of sieved soil per location was air-dried overnight at 40°C in a forced air drying oven. Next, samples were ball milled in steps of 1 minute until samples were visually pulverized to a fine powder. Next, a 9.75-10.25 mg sample was added to tin capsules and squeezed into a tight ball. The sample was analyzed for total carbon (TC) and total nitrogen (TN) using a FlashSmart™ Elemental Analyzer (Thermo Scientific™, Germany) following manufacturer instructions. Two randomly selected samples were double analyzed for TC and TN for method validation

Table S.5 shows the carbon and nitrogen contents, and the carbon to nitrogen (C:N) ratio of each soil sample.

Table S.5: Carbon percentage (C%), nitrogen percentage (N%) and carbon to nitrogen ratio (C:N) of each soil sample. The bottom two rows represent replicates.

Location	C (%)	N (%)	C:N
**SP-I_01**	42.6	1.6	26.7
**SP-I_02**	29.7	1.3	23.1
**SP-I_03**	25.5	1.0	25.3
**SP-I_04**	27.6	1.1	25.1
**SP-I-09**	41.1	1.7	24.7
**SP-I_11**	42.2	1.6	25.9
**SP-I_12**	37.9	1.4	27.7
**SP-I_13**	37.5	1.4	26.0
**SP-I_14**	36.7	1.3	28.4
**SP-I_17**	34.2	1.3	25.4
**SP-I_04 (2)**	21.8	1.1	24.8
**SP-I_09 (2)**	41.0	1.6	24.7

Table S.6 shows the pH values of each soil sample at the start and at the end of the laboratory-based respiration experiment. Table S.7 shows detailed information regarding the added soil and extra additions to each experiment bottle.

Table S.6: pH values for individual soil respiration bottles at the start of the experiment ("Before") and at the end of the experiment ("After"). The difference in pH values is indicated in the "Difference" column, with positive values indicating an increase over time, and negative values indicating a decrease.

Bottle	Before	After	Difference
1	2.9	3.0	0.11
3	6.8	7.2	0.38
5	2.9	3.0	0.10
7	6.9	7.2	0.29
9	3.0	3.0	0.04
11	6.9	7.1	0.25
13	3.0	3.1	0.13
15	7.0	7.2	0.28
17	2.9	3.0	0.06
19	6.9	7.2	0.36
21	3.0	3.0	0.02
23	7.0	7.2	0.21
25	2.9	3.0	0.13
27	6.9	7.2	0.23
29	2.9	2.9	0.02
31	6.9	7.2	0.29
33	2.9	2.9	0.01
35	6.9	7.2	0.27
37	3.0	2.9	-0.03
39	7.0	7.2	0.21

Table S.7: Per location, four treatment bottles are described for their moisture content (%), WHC (%) (for bottles with WHC higher than 60%, moisture and WHC measurements were taken a second time), amount of fresh soil added to the bottle (g), added water (g), glucose (ml) (for bottles 1-17 and then all the glucose test bottles had a second glucose addition), and the amount of added calcium carbonate (g).

Location	Bottle (n)	Moist (%)	Moist 2 (%)	WHC (%)	WHC 2 (%)	Fresh soil (g)	Water (g)	Glucose (ml)	Glucose 2 (ml)	Lime (g)
SP-I_01	1	54.57	-	50.81	-	110.14	11.9	-	1	-
SP-I_01	2	54.57	-	50.81	-	110.75	10.61	1	1	-
SP-I_01	3	54.57	-	50.81	-	110.61	11.9	-	1	24.440
SP-I_01	4	54.57	-	50.81	-	110.47	10.61	1	1	24.416
SP-I_02	5	51.98	-	58.92	-	104.29	2.24	-	1	-
SP-I_02	6	51.98	-	58.92	-	104.30	1.1	1	1	-
SP-I_02	7	51.98	-	58.92	-	104.48	2.11	-	1	21.318
SP-I_02	8	51.98	-	58.92	-	104.16	1.24	1	1	21.280
SP-I_03	9	40.24	-	42.59	-	83.80	14.86	-	1	-
SP-I_03	10	40.24	-	42.59	-	83.37	13.64	1	1	-
SP-I_03	11	40.24	-	42.59	-	83.42	14.86	-	1	19.656
SP-I_03	12	40.24	-	42.59	-	83.34	13.64	1	1	19.596
SP-I_04	13	44.32	-	51.04	-	89.83	8.05	-	1	-
SP-I_04	14	44.32	-	51.04	-	89.99	7.01	1	1	-
SP-I_04	15	44.32	-	51.04	-	89.81	8.05	-	1	19.572
SP-I_04	16	44.32	-	51.04	-	89.75	7.01	1	1	19.552
SP-I_09	17	46.29	-	46.32	-	93.14	13.89	-	-	-
SP-I_09	18	46.29	-	46.32	-	93.25	12.99	1	1	-
SP-I_09	19	46.29	-	46.32	-	93.08	13.89	-	-	21.394
SP-I_09	20	46.29	-	46.32	-	93.25	12.99	1	1	21.248
SP-I_11	21	61.26	58.54	69.01	61.63	129.52	1	-	-	-
SP-I_11	22	61.26	58.54	69.01	61.63	129.23	0	1	1	-
SP-I_11	23	61.26	58.54	69.01	61.63	129.13	1	-	-	26.276
SP-I_11	24	61.26	58.54	69.01	61.63	129.37	0	1	1	26.196
SP-I_12	25	50.33	-	50.79	-	100.16	10.54	-	-	-
SP-I_12	26	50.33	-	50.79	-	100.64	9.2	1	1	-
SP-I_12	27	50.33	-	50.79	-	100.54	10.54	-	-	22.216
SP-I_12	28	50.33	-	50.79	-	100.72	9.94	1	1	22.132
SP-I_13	29	59.60	56.78	71.87	64.01	123.75	1	-	-	-
SP-I_13	30	59.60	56.78	71.87	64.01	123.75	0	1	1	-
SP-I_13	31	59.60	56.78	71.87	64.01	123.75	1	-	-	25.77
SP-I_13	32	59.60	56.78	71.87	64.01	123.75	0	1	1	25.77
SP-I_14	33	51.18	-	56.66	-	102.40	4.05	-	-	-
SP-I_14	34	51.18	-	56.66	-	102.40	3.05	1	1	-
SP-I_14	35	51.18	-	56.66	-	102.40	4.05	-	-	21.29
SP-I_14	36	51.18	-	56.66	-	102.40	3.05	1	1	21.29
SP-I_17	37	45.98	-	50.84	-	92.55	8.65	-	-	-
SP-I_17	38	45.98	-	50.84	-	92.55	7.65	1	1	-
SP-I_17	39	45.98	-	50.84	-	92.55	8.65	-	-	20.24
SP-I_17	40	45.98	-	50.84	-	92.55	7.65	1	1	20.24

References

Berveiller, D., Delpierre, N., Dufrêne, E., Pontailler, J.-Y., Vanbostal, L., Janvier, B., Mottet, L. and Cristinacce, K.: (2005-2014) FLUXNET2015 FR-Fon Fontainebleau-Barbeau, Dataset. https://doi.org/10.18140/FLX/1440161, 2016

Berveiller, D., Dufrêne, E., Delpierre, N., Morfin, A., Vincent, G., Bazot, S., Soudani, K., Girardin, C., Guillot, T., Perot-Guillaume, C.: ETC L2 Fluxnet (half-hourly) from Fontainebleau-Barbeau, 2018-12-31–2024-12-31, ICOS RI, https://doi.org/11676/XYginabgpNEJqxZpreIA2Jct, 2025

Borken, W., Y.-J. Xu, E.A. Davidson and F. Beese: Site and temporal variation of soil respiration in European beech, Norway spruce, and Scots pine forests. Global Change Biology, 8, 1205-1216, https://doi.org/10.1046/j.1365-2486.2002.00547.x, 2002.

Burba, G., D. Anderson, , L. Xu and D. McDermitt: Correcting apparent off-season CO_2 uptake due to surface heating of an open path gas analyzer: progress report of an ongoing study. Proceedings of 27th Annual Conference of Agricultural and Forest Meteorology, San Diego, California, 13pp, 2006.

Burba, G.G., D.K. McDermitt, A. Grelle, D.J. Anderson and L.K. Xu: Addressing the influence of instrument surface heat exchange on the measurements of CO2 flux from open-path gas analyzers. Global Change Biology, 14(8), 1854-1876, https://doi.org/10.1111/j.1365-2486.2008.01606.x, 2008.

Dolman, A., E. Moors, and J. Elbers: The carbon uptake of a mid latitude pine forest growing on sandy soil. Agric. For. Meteorol., 111, 157-170, https://doi.org/10.1016/S0168-1923(02)00024-2, 2002.

Domec, J., Loustau, D., Aluome, C., Chipeaux, C., Denou, J., DEPUYDT, J., Garrigou, C., Kruszewski, A., Lafont, S.: ETC L2 Fluxnet (half-hourly) from Bilos, 2018-12-31–2024-12-31, ICOS RI, https://doi.org/11676/tJ4vI_PK_csVSBxiTr3boJId, 2025

Elbers, J., C. Jacobs, B. Kruijt, W. Jans and E. Moors: Assessing the uncertainty of estimated annual totals of net ecosystem productivity: A practical approach applied to a mid latitude temperate pine forest. Agric. For. Meteorol., 151, 1823-1830, https://doi.org/10.1016/j.agrformet.2011.07.020, 2011.

Feigenwinter, I., Etzold, S., Gharun, M., Hortnagl, L., Meier, P., Liechti, K., Stutz, T., Burri, S., Zweifel, R., Buchmann, N., Baur, T., Eugster, W., Gessler, A., Hug, C., Häni, M., Hüglin, C., Kumar, S., Marty, M., Schmitt Oehler, M., Staudinger, M., Sutter, F., Thimonier Rickenmann, A., Trotsiuk, V., Waldner, P., Wilhelm, M., Zhu, J., Zimmermann, S.: ETC L2 Fluxnet (half-hourly) from Davos, 2018-12-31–2024-12-31, ICOS RI, https://doi.org/11676/5sBqU9dHRiq9i1hHMRCWgGZu, 2025

Foken, T., M. Göckede, M. Mauder, L. Mahrt, B. Amiro, W. Munger (2004) Post-field data quality control. In X. Lee et al. (eds.), Handbook of Micrometeorology, 181-208, 2004.

Goldstein, A.: (1997-2007) FLUXNET2015 US-Blo Blodgett Forest, Dataset. https://doi.org/10.18140/FLX/1440068, 2016

Gough, C., Bohrer, G. and Curtis, P.: (2000-2014) FLUXNET2015 US-UMB Univ. of Mich. Biological Station, Dataset. https://doi.org/10.18140/FLX/1440093, 2016

Heliasz, M., Kljun, N., Biermann, T., Holst, J., Holst, T., Kornacher, P., Linderson, M., Molder, M., Rinne, J.: ETC L2 Fluxnet (half-hourly) from Hyltemossa, 2017-12-31–2024-12-31, ICOS RI, https://doi.org/11676/rnCmH1Z5GgIDXIq4kJ6GdZOb, 2025

Hörtnagl, L., Eugster, W., Buchmann, N., Paul-Limoges, E., Etzold, S., Haeni, M., Pluess, P., and Baur, T.: (2004-2014) FLUXNET2015 CH-Lae Laegern, Dataset. https://doi.org/10.18140/FLX/1440134, 2016

Ibrom, A. and Pilegaard, K.: (1996-2014) FLUXNET2015 DK-Sor Soroe, Dataset. https://doi.org/10.18140/FLX/1440155, 2016

Kljun, N., Molder, M., Lehner, I., Bergström, G., Båth, A., Holst, J., Linderson, M.: ETC L2 Fluxnet (half-hourly) from Norunda, 2017-12-31–2024-12-31, ICOS RI, https://doi.org/11676/epwZ7_H-gvTQ5kb83RnDf6bz, 2025

Knohl,A., Tiedemann, F., Kolle, O., Schulze, E-D., Kutsch, W., Herbst, M. and Siebick, L. (2000-2012) FLUXNET2015 DE-Hai Hainich, Dataset. https://doi.org/10.18140/FLX/1440148, 2016a

Knohl, A. Tiedemann, F., Kolle,O., Schulze, E.-D., Anthoni, P., Kutsch, W., Herbst, M. and Siebicke, L.: (2002-2012) FLUXNET2015 DE-Lnf Leinefelde, Dataset. https://doi.org/10.18140/FLX/1440150, 2016b

Kolari, P., Aalto, P., Keronen, P., Kulmala, L., Kulmala, L., Sahoo, G.: ETC L2 Fluxnet (half-hourly) from Varrio, 2016-12-31–2024-12-31, ICOS RI, https://doi.org/11676/d3obD6InkFMAuYrVCEsbqfOs, 2025

Korkiakoski, M., Aaltonen, H., Aurela, M., Hatakka, J., Laurila, T., Lohila, A., Rainne, J., Tuovinen, J.: ETC L2 Fluxnet (half-hourly) from Lettosuo, 2016-12-31–2024-12-31, ICOS RI, https://doi.org/11676/-b2ChwqUFeve17Xgc6NjvRrU, 2025

Ligne, A., Manise, T., Heinesch, B., Aubinet, M. and Vincke, C.: (1996-2014) FLUXNET2015 BE-Vie Vielsalm, Dataset. https://doi.org/10.18140/FLX/1440130, 2016

Mammarella, I., Keronen, P., Kolari, P., Launiainen, S., Pumpanen, J., Rannik, Ü., Siivola, E., Levula, J., Pohja, T. and Tesala, T.: (1996-2014) FLUXNET2015 FI-Hyy Hyytiala, Dataset. https://doi.org/10.18140/FLX/1440158, 2016

Matteucci, G.: (1996-2014) FLUXNET2015 IT-Col Collelongo, Dataset. https://doi.org/10.18140/FLX/1440167, 2016

Margolis, H.: (2003-2010) FLUXNET2015 CA-Qfo Quebec - Eastern Boreal, Mature Black Spruce, Dataset. https://doi.org/10.18140/FLX/1440045, 2016

Mauder, M., T. Foken, R. Clement, J.A. Elbers, W. Eugster, T. Grünwald, B. Heusinkveld and O. Kolle: Quality control of CarboEurope flux data–Part 2: Inter-comparison of eddy-covariance software. Biogeosciences, 5, https://doi.org/10.5194/bg-5-451-2008, 2008.

Moore, C.J.: Frequency response corrections for eddy correlation systems. Boundary- Layer Meteorology 37: 17-35, 1986.

Munger, J.: (1991-2012) FLUXNET2015 US-Ha1 Harvard Forest EMS Tower (HFR1), Dataset. https://doi.org/10.18140/FLX/1440071, 2016

Novick, K.and Phillips, R.: (1999-2014) FLUXNET2015 US-MMS Morgan Monroe State Forest, Dataset. https://doi.org/10.18140/FLX/1440083, 2016

Ouimette, A.: AmeriFlux FLUXNET-1F US-Ho1 Howland Forest (main tower), Ver. 3-6, AmeriFlux AMP, (Dataset). https://doi.org/10.17190/AMF/2469453, 2024

Rebmann, C., Rebmann, C., Dienstbach, L., Schmidt, P., Wiesen, R., Meis, J., Feldmann, I., Bastos Campos, F., Dejosez, S., Garcia Quiros, I., Gimper, S., Hautmann, D., Hildebrandt, A., Kempka, P., Paasch, S.: ETC L2 Fluxnet (half-hourly) from Hohes Holz, 2018-12-31–2024-12-31, ICOS RI, https://doi.org/11676/cIVUcyMYb_63u3Fyf0RlD7FA, 2025

Reichstein, M., E. Falge, D.Baldocchi, D. Papale, M. Aubinet, P. Berbigier, ... and R. Valentini: On the separation of net ecosystem exchange into assimilation and ecosystem respiration: review and improved algorithm. *Global change biology*, *11*(9), 1424-1439, 2005.

Santana, R.A., C.Q. Dias-Júnior, J. Tóta da Silva, J.D. Fuentes, R. Souza do Vale, E. Gomes Alves, R.M.N. dos Santos, A.O. Manzi: Air turbulence characteristics at multiple sites in and above the Amazon rainforest canopy. Agricultural and Forest Meteorology, 260–261, https://doi.org/10.1016/j.agrformet.2018.05.027, 2018.

Schmidt, M., Bagheri, S., Baltes, U., Becker, N., Dolfus, D., Drüe, C., Graf, A., Kettler, M., Kummer, S., Mattes, J.: ETC L2 Fluxnet (half-hourly) from Wustebach, 2011-12-31–2024-12-31, ICOS RI, https://doi.org/11676/Xq7wZdAPzUg5Iq5rvO6ZO92f, 2025

Schotanus, P., F.T.M. Nieuwstadt and H.A.R. De Bruin: Temperature measurement with sonic anemometer and its application to heat and moisture fluxes, *Boundary Layer Meteorol.*, **26**, 81–93, doi:10.1007/BF00164332, 1983.

van der Molen, M.K., Barten, J., Kruijt, B., Lootens, R., Snellen, H., Zhao, H. (2025). ETC L2 Fluxnet (half-hourly) from Loobos, 2022-12-31–2024-12-31, ICOS RI, https://doi.org/11676/Hz_PRfzpkwrLsuZWhrwzCzDm, 2026a

van der Molen, M.K., Barten, J., Kruijt, B., Lootens, R., Snellen, H., Zhao, H. (2025). ETC L2 Fluxes from Loobos, 2022-12-31–2025-09-30, ICOS RI, https://doi.org/11676/Ww4zYPNKHkBUcjZxNS4CXN1j, 2026b

van der Molen, M.K., Barten, J., Kruijt, B., Lootens, R., Snellen, H., Zhao, H. (2025). ETC L2 Meteo from Loobos, 2022-12-31–2025-09-30, ICOS RI, https://doi.org/11676/7OvfpkiXvumy_vSHQ6ds3Zbo, 2026c

van der Molen, M.K., Barten, J., Kruijt, B., Lootens, R., Snellen, H., Zhao, H. (2026). ETC NRT Fluxes from Loobos, 2025-09-30–2026-03-16, ICOS RI, https://doi.org/11676/wsW2so1FziYDJIaBCh9Yc7G9, 2026d

van der Molen, M.K., Barten, J., Kruijt, B., Lootens, R., Snellen, H., Zhao, H. (2026). ETC NRT Meteo from Loobos, 2025-09-30–2026-03-16, ICOS RI, https://doi.org/11676/tP9bdwzqaVr9Qqak3la5LGXx, 2026e

van der Molen, M.K, van de Sande, M., in 't Zandt, M., Saccomandi, T., Baartman, S., Zhao, H., & Vila, J.: Data supporting Declining Ecosystem Respiration Linked to Nitrogen Deposition: Insights from a 26-Year FLUXNET Record [Data set]. In Global Change Biology (Version 1, Vol. 2026, Number Virtual Issue 'FLUXNET Towards a Better Understanding of Global GHG Fluxes'). Zenodo. https://doi.org/10.5281/zenodo.19065049, 2026f

van der Molen, M.K., H. Snellen, R. Holzinger, J.G.M. Barten, H. Zhao, L. Ganzeveld, W. Peters, M. Krol, J. Vila-Guerau de Arrellano, B. Kruijt: The ICOS Ecosystem Station Loobos: a pine forest site exposed to atmospheric pollution, submitted to Earth System Science Data, 2026.

Varlagin, A., Kurbatova, J. and Vygodskaya, N.: (1998-2014) FLUXNET2015 RU-Fyo Fyodorovskoye, Dataset. https://doi.org/10.18140/FLX/1440183, 2016

Webb, E.K., G.I. Pearman, and R. Leuning: Correction of flux measurements for density effects due to heat and water vapor transfer. Quart. J. Met. Soc. 106: 85-100, 1980.

Zhao, H.: The Loobos ecosystem first tower dataset: meteorology, turbulent fluxes and net ecosystem exchange (1996 to 2021), Dataset, https://doi.org/10.5281/zenodo.15721310, 2025

Zhao, H., Dolman, H., Elbers, J., Jans, W., Kruijt, B., Moors, E., Snellen, H., Vila-Guerau de Arellano, J., Peters, W., Krol, M. C., Hutjes, R., and van der Molen, M.: The Loobos ecosystem first tower dataset: meteorology, turbulent fluxes and net ecosystem exchange (1996 to 2021), Earth Syst. Sci. Data, 18, 2023–2045, https://doi.org/10.5194/essd-18-2023-2026, 2026
